# Linking physiologically-based pharmacokinetic and genome-scale metabolic networks to understand estradiol biology

**DOI:** 10.1186/s12918-017-0520-3

**Published:** 2017-12-15

**Authors:** Joanna H. Sier, Alfred E. Thumser, Nick J. Plant

**Affiliations:** 1 School of Food Science and Nutrition, Faculty of Mathematics and Physical Sciences, University of Leeds, Leeds, LS2 9JT UK; 2 School of Biosciences and Medicine, Faculty of Health and Medical Sciences, University of Surrey, Guildford, GU2 7XH UK; 3 School of Cellular and Molecular Biology, Faculty of Biological Sciences, University of Leeds, Leeds, LS2 9JT UK

**Keywords:** GSMN, PBPK, Multi-scale, Drug-drug interaction, Estrogen, Breast cancer, Physiologically-based pharmacokinetics, Ordinary differential equation, Endocrine disruptor, Liver metabolism, Estrogen receptor

## Abstract

**Background:**

Estrogen is a vital hormone that regulates many biological functions within the body. These include roles in the development of the secondary sexual organs in both sexes, plus uterine angiogenesis and proliferation during the menstrual cycle and pregnancy in women. The varied biological roles of estrogens in human health also make them a therapeutic target for contraception, mitigation of the adverse effects of the menopause, and treatment of estrogen-responsive tumours. In addition, endogenous (e.g. genetic variation) and external (e.g. exposure to estrogen-like chemicals) factors are known to impact estrogen biology. To understand how these multiple factors interact to determine an individual’s response to therapy is complex, and may be best approached through a systems approach.

**Methods:**

We present a physiologically-based pharmacokinetic model (PBPK) of estradiol, and validate it against plasma kinetics in humans following intravenous and oral exposure. We extend this model by replacing the intrinsic clearance term with: a detailed kinetic model of estrogen metabolism in the liver; or, a genome-scale model of liver metabolism. Both models were validated by their ability to reproduce clinical data on estradiol exposure. We hypothesise that the enhanced mechanistic information contained within these models will lead to more robust predictions of the biological phenotype that emerges from the complex interactions between estrogens and the body.

**Results:**

To demonstrate the utility of these models we examine the known drug-drug interactions between phenytoin and oral estradiol. We are able to reproduce the approximate 50% reduction in area under the concentration-time curve for estradiol associated with this interaction. Importantly, the inclusion of a genome-scale metabolic model allows the prediction of this interaction without directly specifying it within the model. In addition, we predict that PXR activation by drugs results in an enhanced ability of the liver to excrete glucose. This has important implications for the relationship between drug treatment and metabolic syndrome.

**Conclusions:**

We demonstrate how the novel coupling of PBPK models with genome-scale metabolic networks has the potential to aid prediction of drug action, including both drug-drug interactions and changes to the metabolic landscape that may predispose an individual to disease development.

**Electronic supplementary material:**

The online version of this article (10.1186/s12918-017-0520-3) contains supplementary material, which is available to authorized users.

## Background

Estradiol is a major endocrine hormone, and is responsible for modulating multiple biological functionalities throughout the human life cycle. Most notably, it plays a central role in development of the reproductive organs, and continue to be important in the reproductive cycle throughout life [1]. During the menstrual cycle and pregnancy estradiol plays a central role in uterine angiogenesis and endometrial proliferation [2, 3]. Plasma concentrations of total estradiol vary through the menstrual cycle (0.1-1 nM: [4, 5]), increasing steadily through pregnancy to reach approximately 100 nM at term [6].

Given the role of estradiol in core body functions, it is perhaps not surprising that its level has been associated with a number of disease states, and that estradiol-mediated biology is a key therapeutic target. Elevated plasma estrogens have been associated with protection against cardiovascular disease [7], but also an increased risk of breast cancer in post-menopausal women; a two-fold increase in estradiol levels being associated with a 1.3-fold increase in relative risk [8]. This carcinogenic effect is probably mediated through a number of mechanisms, including the generation of genotoxic catecholamine metabolites [9] as well as the modulation of cell proliferation and angiogenesis [2, 10].

Pharmacological agents are used to impact on both the normal biological functions of estradiol (e.g. oral contraceptives), and disease processes (e.g. anti-estrogenic cancer chemotherapy). The action of these therapies may be altered through enzyme induction/inhibition elicited by concomitantly administered drugs (e.g. anticonvulsants such as phenytoin), environmental exposure to estrogen-like endocrine disrupting chemicals [11], or ingestion of phyto-estrogens in the diet [12]. In addition to pharmacological impacts on estrogen biology, there are a number of genetic polymorphisms that may be of clinical importance. For example, genetic variants within CYP19 and 17B–HSD, important enzymes in the biosynthetic pathway of estradiol, are associated with different circulating concentrations of estrogen [13, 14]. Likewise, polymorphisms within the degradation pathways have been associated with altered clearance of estrogens and the increased production of the genotoxic catecholamines [15, 16].

Given the complex biological effects of estrogens, both positive and negative, and the potential for gene/drug/environment interactions, the prediction of an individual’s estrogen biological profile is complex. The related disciplines of computational and systems biology have developed to address questions such as this, allowing the examination of large scale, complex networks to predict the emergent biological response [17, 18]. A first stage in the prediction of the biological phenotype elicited by any chemical is the generation of robust disposition models, most commonly as physiologically-based pharmacokinetic models (PBPK [19]), which allow the prediction of concentration-time curves for chemicals within the body. Enhancing these models with mechanistically-detailed models of biologically important hubs will further increase their predictive power [17, 18].

In the current work, we extend the estrogen PBPK model of Plowchalk and Teeguarden [20], increasing the number of physiological compartments from seven to 18. We demonstrate that this extended model is able to reproduce blood concentration-time curves for estradiol in women following intravenous and oral exposures. We expand this model, replacing the fitted intrinsic clearance term with either a fully mechanistic model of estrogen metabolism within the liver, or a genome-scale model of hepatic metabolism. To demonstrate the utility of these models, we examine the impact of concomitant exposure to the anticonvulsant phenytoin on blood estradiol concentrations, demonstrating the ability to identify a clinically important drug-drug interaction (DDIs).

## Methods

### PBPK model structure

**Fig. 1:**
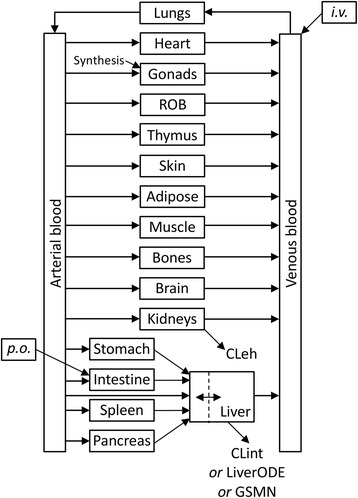
Physiologically based Pharmacokinetic model for estradiol in women. Distribution of estradiol between venous and arterial blood compartments and 16 tissue compartments is represented. The liver and gonads are represented as permeability-limited tissues, and all other compartments well mixed. Estradiol enters the model through synthesis into the gonads, oral dosing (p.o.) into the intestine, and intravenous dosing (i.v.) into the venous blood. Estradiol is removed from the model through extra-hepatic clearance (CLeh) from the kidney, and intrinsic clearance from the liver. Intrinsic clearance is modelled as either a single ODE (Clint), ODE-based model of liver metabolism (LiverODE), or a genome-scale metabolic network (GSMN) as described in text

The PBPK model was created in COPASI v4.14 [21], and was based upon the previously published human female model of Plowchalk and Teeguarden [20]. The model predicts the disposition of estradiol (E2) through 16 tissue compartments plus arterial and venous blood compartments. Estradiol is synthesised within the compartment {gonads}, and subject to extrahepatic clearance (CLeh) and intrinsic clearance (CLint) from the compartments {kidney} and {liver}, respectively. The overall structure of the PBPK model is summarised in Fig. 1.

The original model comprised seven biological compartments: systemic plasma, poorly perfused tissue, richly perfused tissue, pituitary, uterus, ovaries and liver. In addition, an IV input compartment fed into the systemic plasma, while an oral dosing compartment fed into the liver. Here, venous and arterial blood compartments are presented separately, along with 16 tissue compartments, representing the major organs as defined by Peters [22]. Rather than represent uterus and ovaries separately, they are combined in the compartment {gonads}, which represents estradiol target tissues. This reflects a compromise between the desire to predict the distribution of estradiol to target tissues, and the experimental data to validate these predictions. With the exception of the liver and gonads, all compartments are described as well-mixed, rapid equilibrium compartments. The liver and uterus are treated as a permeability-limited compartment with a separate tissue blood compartment [20, 23]. Finally, compartments to represent intravenous (*i.v.*) and oral (*p.o.)* delivery were added to the model, inputting into the venous blood and intestine compartments, respectively.

As estradiol is an endogenous compound, an estrogen biosynthesis reaction was added using mass action kinetics, generating estradiol into the compartment {gonads_cells}. The rate constant for this reaction was fitted to achieve a steady-state blood concentration of 0.15 nM total estradiol, consistent with the published literature [4, 5]. As estradiol in the blood is approximately 98% bound to plasma proteins, this equates to a free plasma concentration of approximately 0.003 nM [24].

An overview of the model structure is presented as Fig. 1, while a full description of reaction parameters, balance equations, global quantities and initial conditions is presented as Additional file 1. Generic physiological parameters were taken from Bosgra [23] and estradiol-specific parameters from Plowchalk and Teeguarden [20]. These values were used to populate ordinary differential equations as described by Peters [22]. The model is available for download from BioModels [25] and www.LiverSystems.org.

### PBPK-LiverODE model structure

A mechanistic model of estrogen metabolism within the liver was created, hereafter denoted as LiverODE (Additional file 1: Figure S3). The model comprises five compartments: an input compartment representing either medium or blood; an output compartment representing medium or bile; and three compartments representing parts of the hepatocyte, namely apical membrane space, basolateral membrane space, and cell interior. Within the input compartment, estradiol is present as E2 and E2free to represent diffusion-limited access of estradiol to cells. E2free represents the bulk of estradiol in the input compartment, while E2 represents estradiol immediately bordering cells that can gain access to the cell membrane and enter cells. This diffusion-limited access is simulated through first order kinetics, with a rate constant based upon previously used estimates for chemical diffusion through medium [26]. Movement between compartments is through either passive diffusion or active transport. Within the hepatocyte, estradiol is subject to sequestration through both specific (i.e. binding to the estrogen receptor) and non-specific binding. Finally, estradiol undergoes a number of metabolic conversions: it is interconverted with estrone through the actions of 17β-hydroxysteroid dehydrogenase 1 and 2 [27, 28]; both estradiol and estrone are metabolised via CYP1A1 and CYP3A4 during Phase I metabolism to form hydroxylated metabolites [29, 30]; the catecholamine metabolites from these reactions are potent mutagens, and are readily deactivated through the action of catechol-O-methyltransferase (COMT) [31]; finally, both estradiol and estrone are metabolised via SULT1E1, SULT2A1, UGT1A1, UGT1A3 and UGT2B7 during Phase II metabolism [32] to form sulphate and glucoronide conjugates, respectively. These interconversions were selected as they represent the major metabolic fates of estradiol in the liver, and robust parameter values were available within the literature. The LiverODE model was used to replace the intrinsic clearance term (Cl_int_) of the base PBPK model by merging of the {liver_plasma} compartment of the PBPK model and the {medium} compartment of the LiverODE model. This model is hereafter designated the PBPK-LiverODE model. The model was validated against in vitro data for estradiol clearance in primary human hepatocytes (Additional file 1: Figure S4).

A full description, including overall structure, reaction parameters, balance equations, global quantities and initial conditions, of the model is presented as Additional file 1. The model is available for download from BioModels [25] and www.LiverSystems.org.

### PBPK-GSMN model structure

The PBPK-GSMN model is based upon the PBPK model, but with the Cl_int_ term replaced by a genome scale metabolic network (GSMN). Briefly, GSMNs capture the total connectivity of a metabolic network within a stoichiometric matrix, and coupled with constraint based modelling approaches allow the examination of metabolite flow through the entire network [18, 33]. Here, we used the Recon2 GSMN, the most comprehensive general reconstruction of human metabolism, comprising 7440 reactions and 5764 metabolites [34].

To decrease the solution space for constraint based modelling approaches additional constraints can be added, increasing the robustness of predictions. Each reaction within the GSMN is constrained by an upper bound (UB) and lower bound (LB), representing the maximum and minimum flux, respectively. Within Recon2, the maximum value for a bound is 1000, with directionality reflected in the sign of this value. Hence, a unidirectional forward reaction is defined as 0,1000 (LB, UB), a unidirectional reverse reaction (−1000,0), and a bidirectional reaction (−1000,1000). The LB and UB can be reduced to further constrain the solution space where additional biological information is present. Here, these bounds were further constrained in two ways. First, the physiological import-physiological export set was used (PIPES; [35]), which only permits exchange of metabolites normally present under physiological conditions. The LB and UB for exchange reactions present within PIPES were set to −1 and 1000, respectively, reflecting limited consumption but unlimited production of these metabolites by the cell. For all exchange reactions not present within PIPES, UB and LB values were set to 0. Second, PIPES exchange reaction bounds were further constrained through experimentally-derived nutrient production and consumption rates, where available [36]. Jain et al. determined the consumption and production rates for 219 metabolites across the NCI60 cell culture collection. We assume that the maximal values for consumption of each metabolite across these immortal cell lines at least equals, and most likely exceeds, that observed in vivo. We can therefore use these values to set the LB for exchange reactions within the PIPES. For example, the maximal rate of glucose depletion from the medium across the NCI60 cell culture collection was −1.723855 mmol/gDW/h, reflecting the maximal consumption rate by cells. On this basis, the exchange reaction for glucose (Recon2 ID: R_EX_glc_LPAREN_e_RPAREN) was constrained with lower and upper bounds of −1.723855 and 1000, respectively.

Traditionally, constraint-based modelling approaches have used the Biomass Reaction as an objective function (R_Biomass_Reaction), which represents those constituents required for cell division (e.g. ATP, amino acids, nucleotides etc.) and thus can be used as a surrogate for cell division [33].. While this is appropriate for many prokaryotic models, where a main objective is to grow, it is inappropriate for many eukaryotic cells. For example, liver cells are effectively senescent, but do retain a significant regenerative capacity following liver injury. To represent this regenerative capacity, a ‘Liver turnover’ constraint node was added, which requires a minimum flux through the Biomass Reaction (0.007). This further constrains the solution space to represent the potential for cell division, but does not require the simulation to optimise for cell growth. Two objective functions for the flux balance analysis of the GSMN are used within the current work, representing homeostatic functions of the liver. First, we explore glucose-lactate homeostasis: Lactate is constantly produced by red blood cells through respiration, but blood levels must be maintained to prevent acidosis. The liver consumes lactate and converts it to glucose, which is reflected by setting optimisation of external glucose production as the first objective function. Glucose production (R_EX_glc_ LPAREN_e_RPAREN_) and lactate consumption (R_EX_lac_L_LPAREN_e_RPAREN_) fluxes are extracted from an example flux distribution predicted by flux balance analysis and used to predict blood concentrations of lactate and glucose. Second, the role of the liver in estrogen and urea metabolism is explored: Estrogen is an important endocrine signalling molecule, while ammonia is produced through the metabolism of amino acids throughout the body, and converted to urea in the liver for subsequent elimination through the urine. Estradiol secretion is set as the second objective function, representing the intrinsic clearance of estradiol by the liver. Fluxes of interest are read from an example flux distribution predicted by flux balance analysis: excretion of urea (Recon2 ID: R_EX_Urea_LPAREN_e_RPAREN_), excretion of estradiol (Recon2 ID: R_EX_estradiol_LPAREN_e_RPAREN), formation of estrone sulphate (Recon2 ID: R_ESTSULT), and formation of 2-OH estradiol (Recon2 ID: R_RE3013R). Flux toward urea and estradiol excretion are used to predict blood concentrations, flux toward estrone sulphate measures a major metabolic fate of estradiol, and flux toward 2-OH estradiol represents catecholamine formation [33].

The PBPK and GSMN parts of the model were coupled through the species ‘E2{liver_cells}’, representing free estradiol within the hepatocyte. The model was constructed in MuFINS [37], in which ODE-based networks are represented in Petri net (PN) formalism and linked to the GSMN through special directed arcs using our quasi steady-state Petri net (QSSPN) approach [38]. This allows connection of metabolite (fluxes) between the PN and GSMN parts of the model, as well as the setting of flux bounds within the GSMN by the PN part of the model.

An advantage of the modelling approach used here is the ability to dynamically alter flux bounds within the GSMN [37]. This allows, for example, the exploration of the impact of drug action on global metabolism. Here, we model drug-drug interactions occurring through activation of the pregnane X-receptor (PXR). PXR is a member of the nuclear receptor superfamily, and is activated by a large range of both endogenous and exogenous chemicals, altering the expression of metabolic enzymes and membrane transporters to respond to the stimulus [39]. Drug interaction with PXR is represented by mass action kinetics, with kon and koff rates set to reproduce the experimentally determined affinity. Binding of an agonist with PXR forms PXRL, the active form of PXR, which increases the transcription of PXR target genes. Predicted concentration of the encoded protein species vary from 111 nM (basal transcription, no PXRL present) to 518 nM (maximal transcription, maximal level of PXRL). These protein concentrations are used to inform the flux bounds for mapped reactions [37, 38], with the Petri net place representing the encoded protein containing a comment that sets the upper and lower reaction flux bounds for different predicted protein concentrations: these range from 0,0.23 (lower bound, upper bound) at 100 nM protein to 0,0.63 at 550 nM protein, which are values that reproduce the known rates of metabolism of estradiol in primary human hepatocytes (Additional file 1).

PXR target genes were identified through a two-step process: First, transcriptomic data from the Japanese Toxicogenomics Program for primary human hepatocytes exposed to 2.8μM, 14μM and 70μM of the classical PXR ligand rifampicin for 2, 8 and 24 h [40] were extracted from ArrayExpress (E-MTAB-798) [41]. Array analysis was performed within the Bioconductor R suite with data pre-processing using *affy*, and differential gene expression using *limma*. Second, the list of differentially expressed genes was further refined to include only direct targets of PXR. These genes were identified through literature evidence of a direct interaction via chromatin immunoprecipitation.

A cartoon of this model is presented as Additional file 1: Figure S5, with a full description, including overall structure, reaction parameters, global quantities, initial conditions, and literature evidence for PXR target genes also presented as Additional file 1.

### Virtual patient population

A virtual population was generated using the National Health and Nutrition Examination Survey (NHANES) 2013–14 anthropometric data [42], representing a modern U.S. population. Briefly, anthropometric data was extracted for 1495 women aged 18–45 years inclusive. Individual weight (Kg) and height (m) data are used to calculate body mass index [weight/height^2^] and body surface area [0.00718*weight^0.425^*(height*100)^0.725^]. These four parameters are used to predict individual organ weights and blood flows using the equations presented in Bosgra et al. [23]. Summary statistics for the NHANES cohort are presented as Additional file 1.

### Model simulation and analysis

Both the PBPK and PBPK-LiverODE models were simulated in COPASI [21], while the PBPK-GSMN model was simulated using MuFINS [37]. Statistical analysis was undertaken using GraphPad Prism v6.01 (GraphPad Software Inc., La Jolla, USA). Datasets were compared through a two-way ANOVA with Sidak’s multiple comparison test. The level of statistical significance was set a priori at *p* < 0.05.

## Results and discussion

### Validation of models against clinical data

We first explored the ability of the three models to reproduce known human responses to estradiol exposure. First, we examined the ability of the models to reproduce the binding of estradiol to plasma proteins, specifically albumin and steroid hormone binding protein (SHBG). Second, we predicted the concentration-time curve for plasma estradiol in humans following *i.v.* dosing, and compared this to in vivo data. Third, concentrations time curves for oral dosing were compared to human data. Fourth, we explored how predicted compartment dispositions may reflect the in vivo situation.

**Fig. 2:**
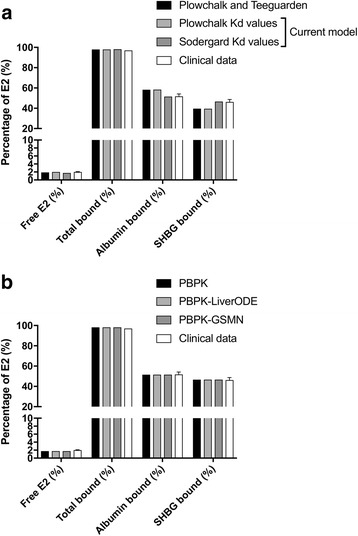
Prediction of estradiol binding to plasma proteins. **a** Steady-state binding of 25 nM estradiol to albumin and steroid hormone binding globulin (SHBG) were simulated within the PBPK model and compared to clinical data and the prediction of Plowchalk and Teeguarden. **b** Steady-state binding of 25 nM estradiol to albumin and SHBG were simulated for the PBPK model and compared to prediction from the PBPK-LiverODE and PBPK-GSMN models, and experimental data of Sodergard et al. [43]

Within the plasma compartment, the model predicts concentrations of free estradiol, plus estradiol bound to albumin and SHBG. Using a concentration of 25 nM estradiol, the distribution of estradiol within the plasma were predicted for the original Plowchalk and Teeguarden PBPK model and our expanded PBPK model (Fig. 2a). Modelled free fractions are consistent between the two models (approximately 2%), and equivalent to the experimental data of Sodergard et al. [43]. The predicted distribution of plasma protein bound estradiol between albumin and SHBG is also reproduced between the two models (approximately 58% albumin-bound and 40% SHBG-bound), but differs from the clinical data, which indicates approximately 52% of estradiol is albumin-bound and 46% SHBG-bound. In the Plowchalk and Teeguarden model, disassociation constants of 17 μM and 1.5 nM were used for the interaction of estradiol with albumin and SHBG, respectively. In contrast, the work of Sodergard et al. reports values of 23.8 μM and 3.18 nM, respectively [43]. When these values are used, free estradiol remains at approximately 2%, but the resultant distribution of bound estradiol is consistent with the clinical data [44]. These latter values were adopted within the PBPK model. When the PBPK model was expanded to include the mechanistic model of estradiol metabolism (PBPK-LiverODE) or the GSMN (PBPK-GSMN) these percentage distributions were maintained (Fig. 2b).

**Fig. 3:**
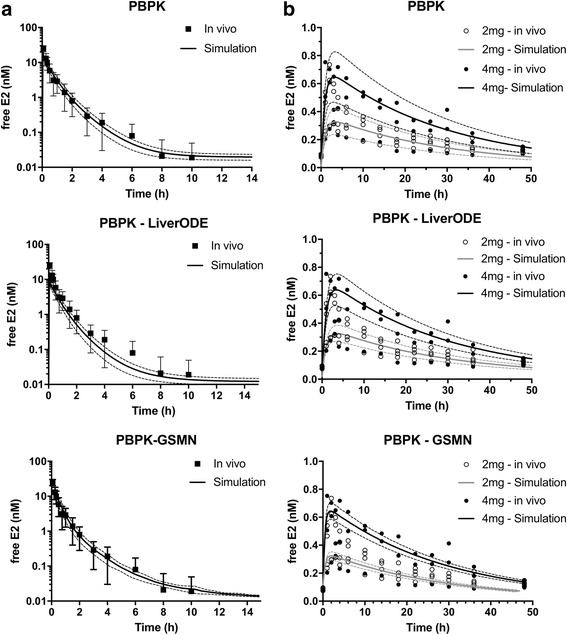
Prediction of estradiol kinetics in women. Estradiol venous blood concentrations were simulated for the 1495-person virtual population following **a** 0.3 mg *i.v.* bolus and **b** 2 mg or 4 mg oral bolus exposures, and presented as mean (±s.d.). The *i.v.* bolus data was compared to clinical data from Kuhnz et al. [45], which is shown as mean (±s.d.), while oral dose simulations were compared to clinical data from Lyrenas et al. [47]

Estrogens are clinically administered through a number of routes, including intravenous, subcutaneous, oral, dermal and rectal. Here, *i.v.* and oral exposure routes have been modelled, and validated against in vivo data. Kuhnz et al. measured plasma levels of estradiol in young women following intravenous administration of a 0.3 mg bolus [45]. Figure 3a, presents this in vivo data, plus simulations for the virtual patient population. In each model, the 0.3 mg bolus was represented by an initial 1101nmoles of estradiol in the {dose_IV} compartment. This was delivered to the {plasma_venous} compartment through a first order mass action kinetics with a rate constant of 250 h^−1^, which was essentially complete within 3 min. Entry to the liver is permeability-limited, with a permeability rate of 1000 L.h^−1^ being used in the original of Plowchalk and Teeguarden paper [20]. For the base PBPK model and the PBPK-GSMN model this was replaced by uptake and efflux rate constants of 1000 L.h^−1^ and 277.8 L.h^−1^, respectively, reflecting a plasma:tissue coefficient of 3.6 [46]. For the PBPK-LiverODE model, it was necessary to vary these constants to best fit the clinical data, while maintaining a plasma:tissue coefficient of 3.6: values of 150 L.h^−1^ and 41.7 L.h^−1^ were used, for uptake and efflux respectively. This represents a 6.7-fold difference in the magnitude of the rate constants between the two model systems. The reason underlying this difference is unclear but may reflect parameter robustness in the LiverODE model. Kinetic parameters are derived from multiple in vitro systems, while protein concentrations are measured using different technical approaches. The parameters derived from these approaches will each have their own precision level, and the sum of these errors will influence the intrinsic clearance of estradiol from the liver. As demonstrated in Fig. 3a, all models were able to reproduce the clinical data with a good degree of accuracy. Root mean square error (RMSE) values decrease across the models, with RMSE values of 3.7, 1.2 and 0.9 for the PBPK, PBPK-LiverODE and PBPK-GSMN models, respectively. The decrease in RMSE value across the models is primarily driven by under prediction at higher concentrations in the base PBPK model.

The ability of the models to reproduce oral dosing was examined next. Lyrenas et al., exposed women to 2 mg and 4 mg of radiolabelled estradiol, and then measured plasma estradiol over the subsequent 50 h [47]. In each model, the estradiol dose was represented by an initial 7342nmoles or 14685nmoles of estradiol (2 mg and 4 mg, respectively) in the {dose_oral} compartment, and delivered to the {intestine} compartment through a first order mass action kinetics with a rate constant of 0.025 h^−1^. In addition to absorption into the intestine, estradiol within the {dose_oral} compartment was depleted with a first order rate constant of 0.01 h^−1^ to represent loss through excretion. These values are consistent with the values used by Plowchalk and Teeguarden [20]. As presented in Fig. 3b, all three models are able to reproduce the clinical data, demonstrating an initial rising phase comprising drug absorption and clearance followed by a declining phase comprising clearance only. Root mean square error (RMSE) values decrease across the models, with RMSE values 2 mg/4 mg oral dose of 0.113/0.175, 0.112/0.174 and 0.110/0.164 for the PBPK, PBPK-LiverODE and PBPK-GSMN models, respectively. The decrease in RMSE value across the models is, again, primarily driven by under prediction at higher concentrations in the base PBPK model. However, we note that there is considerable variability in the clinical samples and as such comments on data fit must be treated with caution.

To further explore the model performance, we calculated the maximal concentration (Cmax), time to Cmax (Tmax) and the area under the concentration-time curve (AUC_∞_) for each simulation (±s.d.) and compared them to the respective in vivo data (Table 1). Prediction of AUC for an i.v. bolus was extremely good for the base PBPK model, and slightly under predicted by both the PBP-LiverODE and PBPK-GSMN models. This under-prediction is driven by under prediction of E2 plasma concentration during the first 30 min after dosing. With regards to oral exposures, we note that the model of Plowchalk and Teeguraden was unable to accurately reproduce oral dose data, with the Tmax values off by several hours [20]. All three models presented here produce good predictions of AUC, Tmax and Cmax following a 2 mg oral dose when compared to the in vivo data of Lyrenas [47]. Simulations of the response to a 4 mg oral dose of estradiol over predict with regard to Cmax and AUC, although prediction of Tmax is robust. However, examination of the in vivo data shows one individual whose response to 4 mg oral estradiol is markedly different, being closer to the response of those individuals exposed to 2 mg oral estradiol. If this individual is removed, then model prediction is within the error of the in vivo data for both Cmax and AUC.

**Table 1:** Pharmacokinetic parameters for three models

		In vivo	PBPK	PBPK-LiverODE	PBPK-GSMN
i.v. – 0.3 mg	AUC	10.2 ± 1.18	10.4 ± 0.23	6.1 ± 0.17	5.9 ± 0.56
Oral – 2 mg	Tmax	3.8 ± 0.4	2.5 ± 0.34	3.5 ± 0.38	2.4 ± 0.18
	Cmax	0.44 ± 0.24	0.34 ± 0.19	0.35 ± 0.24	0.32 ± 0.23
	AUC	10.3 ± 1.18	8.9 ± 0.19	9.2 ± 0.13	8.1 ± 0.04
Oral – 4 mg	Tmax	3.4 ± 0.6	3.1 ± 0.31	3.2 ± 0.35	2.3 ± 0.26
	Cmax	0.63 ± 0.3	0.67 ± 0.25	0.71 ± 0.26	0.65 ± 0.21
	AUC	13.4 ± 3.47	17.3 ± 0.38	17.4 ± 0.24	16.2 ± 0.09

**Fig. 4:**
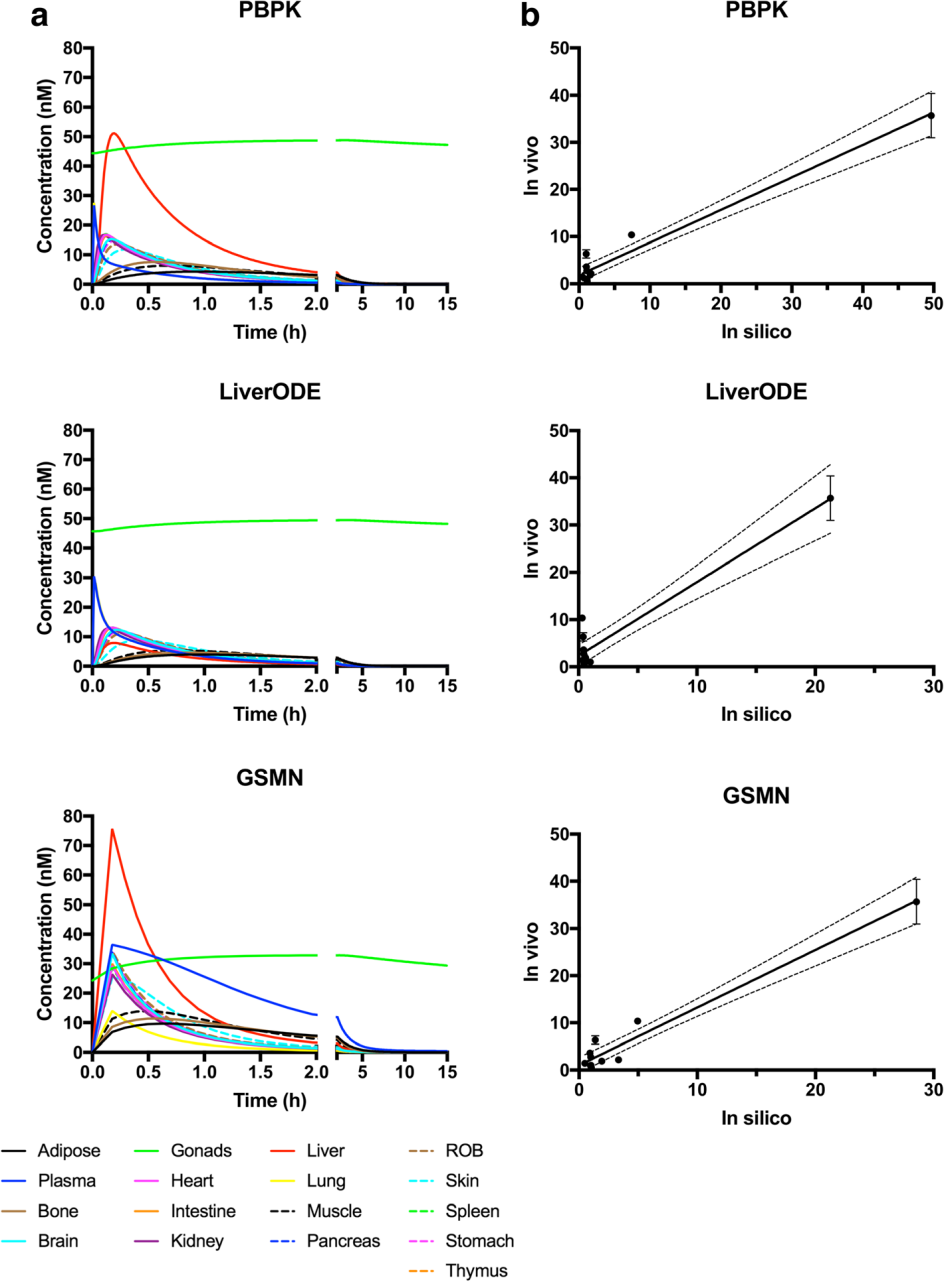
Prediction of estradiol organ distribution. **a** estradiol organ concentrations were simulated over 15 hours following a 0.3mg i.v. bolus. **b** Predicted organ concentrations 1 hour following a 0.3mg i.v. bolus are compared to experimental data from the mouse, presented as mean (+/- s.d.) [48]

In addition to predicting estradiol plasma concentration, the model predicts concentration-time curves for estradiol disposition into 16 tissue compartments. As shown in Fig. 4a, IV dosing with 0.3 mg estradiol is predicted to increase concentrations in each compartment at different rates, with Tmax values varying between 0.1 h {kidney} and 0.93 h {adipose} for the base PBPK model. Clearance of estradiol through the kidney (CLeh) and liver (CLint) results in a decrease in organ concentrations that is essentially complete by 15 h. The distribution to, and clearance from, each organ is qualitatively similar between the three models, but there are quantitative differences. For example, predicted Cmax values for the {liver} compartment is 50.3 nM, 7.9 nM and 75.7 nM for the PBPK, PBPK-LiverODE and PBPK-GSMN models, respectively. We note that the fold-difference in Cmax between the LiverODE model and PBPK (6.4) or PBPK-GSMN (9.6) is consistent with the difference in permeability-limited rate constants between these systems (6.7-fold). Comparison of predicted organ concentration against in vivo data is difficult due a lack of robust in vivo data. Human data is not available, but studies in rodents have been undertaken. Fig. 4b presents correlations between the predictions from each of the models and the mouse disposition data of Benard et al., where disposition of [18F]fluorinated-E2 was examined 1-h after *i.v.* dosing [48]. All three models successfully predict organ concentrations: RMSE is 4.9, 5.5, 3.4 for the PBPK, PBPK-LiverODE and PBPK-GSMN models, respectively. The largest source of variation is in the prediction of E2 concentration within the {gonads} compartment. However, we note the substantial variation in the literature for this measurement, with plasma ratios ranging between 10 and 133. [48–51]. In the current study the ratio is 49.7, 21.3 and 28.5 for the PBPK, PBPK-LiverODE and PBPK-GSMN models, respectively, and as such is consistent with the general literature.

As described in the preceding paragraphs, all three models perform acceptably using a range of metrics. They are able to reproduce with an acceptable degree of accuracy both clinical data and the predictions of the other models. Where there are differences between the model predictions, this may represent the different parameter sets used within the models themselves or the modelling framework used. Clearance from the liver is represented by either a single ODE (CLint), a more comprehensive ODE-based model of estradiol metabolism in the liver, or a genome-scale metabolic network. In each case, the representation of biology has its uncertainties based around model reduction, parameter robustness etc. In addition, the PBPK and PBPK-ODE models are simulated within COPASI, while the PBPK-GSMN model is simulated in MuFINS. This may lead to differences in prediction due to the modelling software. Extensive comparison of model simulations in COPASI and MuFINS suggests that overall model behaviours and the magnitude of observed changes are consistent, but that the exact timing of events may vary. This is most likely due to the application of PNs in the MuFINS software, whereby reactions are simulated through a propensity function that describes the probability that any given transition will fire. Hence, for any given model the same behaviour will occur regardless of the software architecture, but the exact timing of an event may vary.

### Impact of drug exposure on the metabolic phenotype

An important use of in silico models in drug development is the prediction of interactions. Interaction with therapeutic agents, exogenous chemicals (e.g. food components/contaminants), or endogenous chemicals may lead to a reduction in drug efficacy or increased risk of toxicity. In addition, the metabolic adaptation that can occur during chronic drug exposure may predispose individuals to an increased risk of disease development/progression. Here, we explore the impact of chronic phenytoin exposure on two endpoints; the clinically-relevant interaction with estradiol, and the impact on glucose secretion capacity in the liver.

The use of estrogens as both contraceptives and to treat symptoms of the menopause means that individuals are likely to be exposed for chronic periods. This raises the possibility of concomitant exposure to other drugs, and hence the potential for drug-drug interactions. Traditionally, modelling drug-drug interactions has involved a priori knowledge of the interaction, with or without any underpinning mechanistic information. The use of a genome-scale metabolic network has the advantage that metabolite fluxes are based upon known network connectivity and the production/consumption of substrates/cofactors. As such, drug-drug interactions should emerge from the simulation as a result of altered fluxes within the metabolic network, without a priori knowledge of the interaction [37]. This may improve the ability to predict potential DDIs early in drug development, allowing risk-benefit scenarios to be considered.

**Fig. 5:**
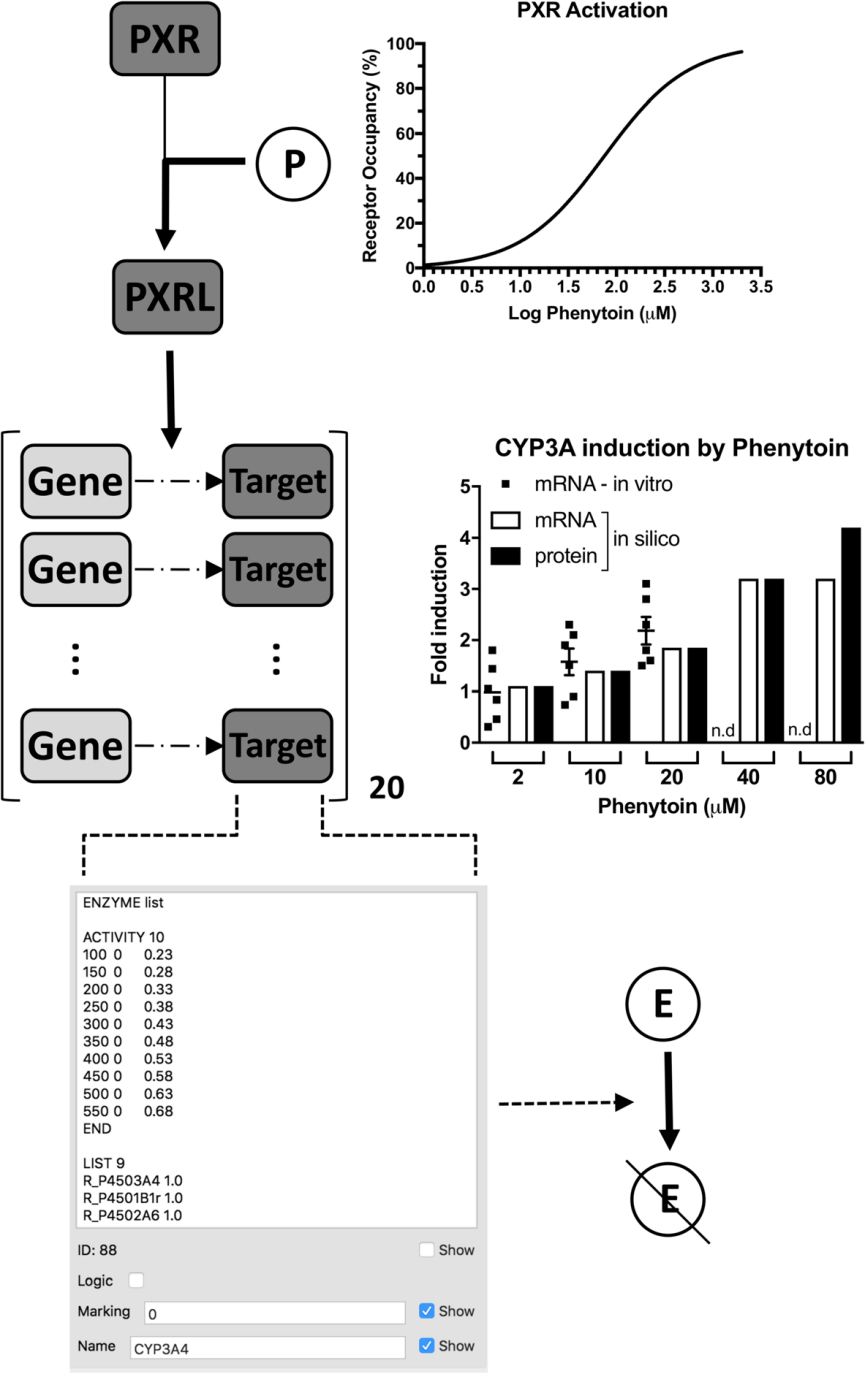
Activation of PXR-mediated gene expression. To represent drug-drug interactions, a kinetic model of PXR-mediated gene expression was generated, based upon Kolodkin et al. [61]. The nuclear receptor PXR is activated by the low affinity ligand Phenytoin (P), forming PXRL. This increases transcription of 20 PXR target genes, leading to increased concentrations of the encoded target proteins. Predicted fold-inductions of CYP3A mRNA and protein are consistent with data from Luo et al. [58]. n.d. = not determined. These target proteins are mapped to reactions within the Recon2 genome scale metabolic network, with their concentration setting reaction bounds within the network. This results in an altered metabolic capacity within the network, leading to increased estradiol (E) metabolism

One scenario where clinically important drug-drug interactions have been identified is during the concomitant use of estrogens and antiepileptic drugs [52, 53]: this scenario is especially important given the teratogenic liability of several anticonvulsant drugs [54]. Nearly one-fifth of fertile women with epilepsy use oral contraceptives, only slightly lower that than the overall population average of 25%, meaning that this is a significant consideration for drug prescription [55]. To demonstrate how a GSMN may aid prediction of DDIs we extended the base PBPK-GSMN model to include PXR-mediated induction of gene expression. PXR is a member of the nuclear receptor family of ligand activated transcription factors and regulates the gene expression of a range of metabolic enzymes [39, 56]. The anticonvulsant phenytoin is a low affinity ligand for PXR [39, 57, 58], and activates the expression of a range of proteins involved in the metabolism of estrogens [39, 56]. As such, activation of PXR is the mechanistic underpinning for the phenytoin-estradiol DDI, as well as a large number of other clinically important DDIs [59, 60]. A module representing the activation of PXR by phenytoin, plus the subsequent transcription and translation of 20 target genes was developed, and is depicted in Fig. 5. These target genes include phase I metabolic enzymes (e.g. CYP3A4), phase II metabolic enzymes (e.g. GSTA1), and drug transporters (e.g. ABBC3). While this is not an exhaustive list of all direct PXR target genes, and excludes indirect target genes and inhibition of expression, it is interesting to note that it maps to 173/7440 (2.3%) reactions within Recon2. As detailed in the Additional file 1, where possible kinetic parameters are based upon literature values and have been previously published [61]. We note that we use an approach analogous to PBPK model construction, whereby a generic model may be tailored through the use of chemical-specific parameters. Here, we use a previously published ODE-based model of the transcriptional effects of PXR, with rifampicin as the modelled ligand [61]. This model is altered to represent phenytoin-mediated induction by changing the parameters representing Kd between ligand and PXR. Such an approach presumes that the downstream effects of PXR activation are analogous between the two ligands, a reasonable starting assumption unless evidence exists to the contrary. Predictions from the resultant model were compared to the experimental data of [58].

Exposure of cells to 2, 10 or 20μM phenytoin results in a fold induction (±s.d.) of 0.9 (±0.26), 1.6 (±0.29) and 2.2 (±0.25) in CYP3A mRNA, respectively. As shown in Fig. 5, these values are consistent with fold increases predicted by the model. Under normal therapeutic use, the steady-state blood concentration of phenytoin is in the range 10-20μg/ml, which is equivalent to 40–80μM. We therefore undertook simulations under these conditions, with CYP3A transcripts predicted to increase from 0.067 nM to 0.21 nM and 0.28 nM, while concentration of the encoded protein is predicted to rise from 111 nM to 351.5 nM and 467.9 nM, respectively; these are equivalent to 3.2–fold and 4.2–fold inductions. This increase in protein expression is reflected in the GSMN through altered bounds for all mapped reactions. For example, the CYP3A4 protein is mapped to nine reactions within the Recon2 GSMN, including the metabolic conversion of estradiol to its catecholamine metabolites (R_RE3013C and R_RE3013R). Under basal conditions, where CYP3A4 protein concentration is 111 nM, flux bounds for these reactions are 0,0.23 (LB < UB), reflecting an irreversible reaction. Activation of PXR results, and thus increased CYP3A4 protein levels, increases the UB to 0.48 and 0.58 at 351 nM and 467 nM, respectively (Fig. 5). It should be noted that this increases the bounds of the reactions, but does not necessarily increase flux through these reactions; this will only occur if the previous bound was limiting with respect to maximisation of the objective function. In the case of estradiol metabolism, these flux bounds are limiting, as the predicted flux for metabolism of estradiol by CYP3A4 increases from 0.23 to 0.48 and 0.58 as the bounds increase (Additional file 1: Figure S6).

**Fig. 6:**
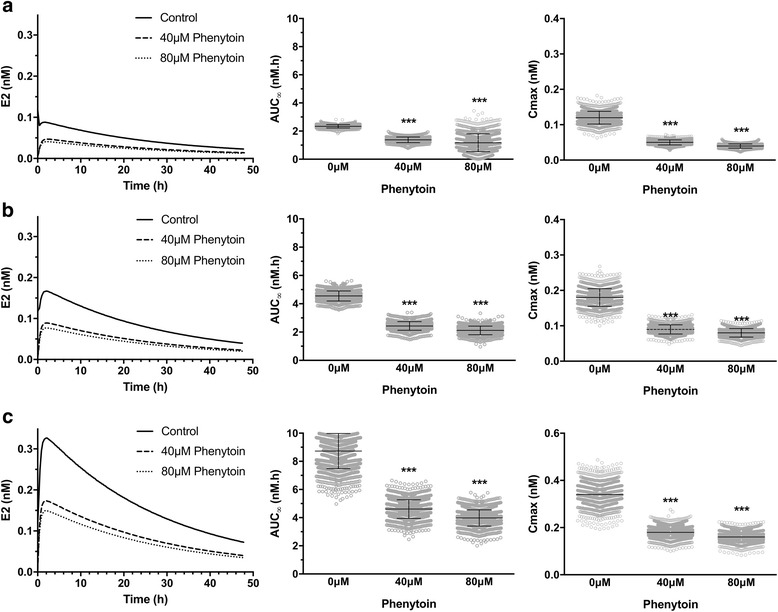
Impact of phenytoin on single oral dose estradiol kinetics in women. Estradiol venous blood concentrations were simulated for the 1495-person virtual population following **a** 0.5 mg, **b** 1 mg, or **c** 2 mg oral estradiol. PXR occupancy, PXR target-gene mRNA and protein levels were set to reflect concomitant exposure to 0μM, 40μM or 80μM phenytoin, as indicated. A representative concentration-time plot is shown in the left panel, with AUC and Cmax (mean ± s.d.) presented in the middle and right panels, respectively. Statistical significance was assessed using a two-way ANOVA with Sidak’s multiple comparison test. *** = *p* < 0.001 compared to 0μM phenytoin

The impact of concomitant exposure to phenytoin and oral estradiol is shown in Fig. 6. Simulations are carried out assuming chronic administration of phenytoin, meaning that phenytoin-mediated changes in protein levels have reached a steady-state. Initial levels of PXR target genes (mRNA and protein) are set to 0.21/351.5 nM and 0.28/467.9 nM for 40μM and 80μM phenytoin scenarios, respectively, compared to 0.067 /111 nM in the non-induced condition. Initially, we examined the impact of phenytoin co-exposure on the response to a single oral dose of estradiol. Estradiol is commonly prescribed at doses equivalent to 0.5 mg/day, 1 mg/day and 2 mg/day, and at all three dose levels concomitant exposure to phenytoin is predicted to alter estradiol kinetics (Fig. 6). Tmax values do not alter, remaining at just over two hours, but there are significant decreases in Cmax and AUC. On average, estradiol plasma AUC values decrease to 56%, 54% and 53% (0.5 mg, 1 mg and 2 mg estradiol, respectively) with concomitant exposure to 40μM phenytoin. These further decrease to 47%, 47% and 46% with 80μM phenytoin. These values are consistent with the approximate 50% decrease reported in the literature [52, 62].

**Fig. 7:**
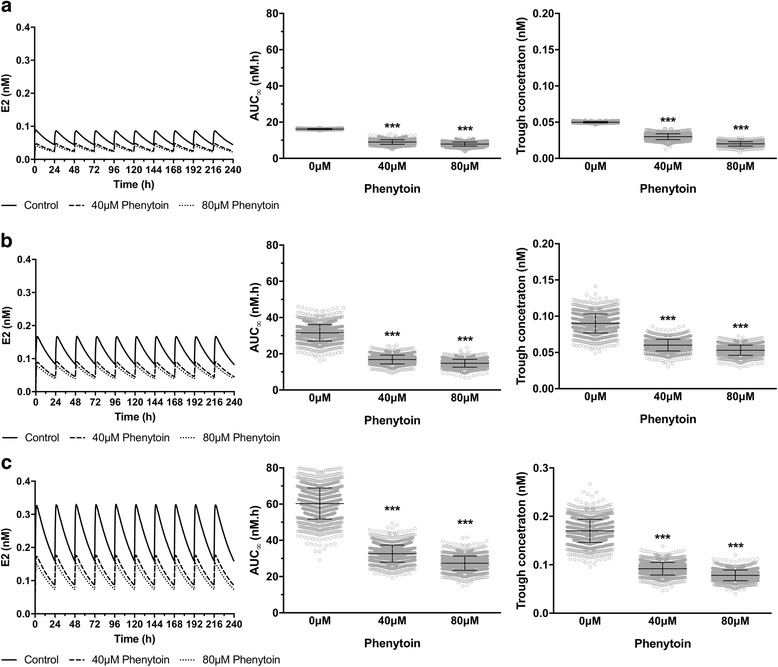
Impact of phenytoin on repeat oral dose estradiol kinetics in women. Estradiol venous blood concentrations were simulated for the 1495-person virtual population following **a** 0.5 mg, **b** 1 mg, or **c** 2 mg oral estradiol. A representative concentration-time plot is shown in the left panel, with AUC and trough concentration (mean ± s.d) presented in the middle and right panels, respectively. Statistical significance was assessed using a two-way ANOVA with Sidak’s multiple comparison test. *** = *p* < 0.001 compared to 0μM phenytoin

Estradiol is most likely to be prescribed over a chronic period of time when used as an oral contraceptive. We therefore examined the impact of concomitant phenytoin exposure on such a scenario. As before, initial conditions of PXR activation and target gene expression were set for 0μM, 40μM and 80μM phenytoin to reflect an individual already on therapy. Simulations were then undertaken for a 10-day period where estradiol was administered as an oral dose of 0.5 mg/day, 1 mg/day or 2 mg/day. As shown in Fig. 7, a once-a-day dosing regime is sufficient to achieve a steady state exposure to estradiol. There is a significant intra-day variability, with peak and trough concentrations varying by approximately 2-fold for all exposures. Concomitant exposure to phenytoin is predicted to produce a similar fold-decrease in AUC as seen for the single oral dose, being approximately 55% and 47% of the control value for 40μM and 80μM phenytoin, respectively. Trough plasma estradiol concentrations are also predicted to decrease by a similar level (Fig. 7).

The impact of chronic drug exposure is unlikely to be limited to drug-drug interactions, with a current challenge being to predict the potential long-term adverse health impacts of such chronic exposure. To explore this, we examined sample steady-state flux distributions within the GSMN under both basal (i.e. no phenytoin) and 80μM phenytoin exposure scenarios. Under basal conditions, a maximal rate of glucose secretion of 6.8 mmole/g dry weight/h was predicted. In contrast, chronic exposure to 80μM phenytoin was predicted to increase this to 7.2 mmole/g dry weight/h. A full list of reaction fluxes for each scenario is provided as Additional file 2: 1032 reactions are different between the two sample solutions, representing approximately 14% of the total reaction in the GSMN. It should be noted, however, that some of these differences are likely to represent technical artefacts where the solver can identify two flux distributions that reach the same optimal solution. These alternate solutions may represent the known redundancy in biological networks, which contributes to the overall robustness of the system. Regardless, this supports the notion that drugs can have large-scale impacts on metabolic systems. It should be noted that while we have used phenytoin as the drug in this example, as the driver for metabolic adaptation is activation of PXR then any PXR agonist could cause these effects. Nuclear receptors, most notably PPARs, have been linked to energy metabolism within the body, but increasing evidence supports the role of a number of other nuclear receptors [63, 64]. Increased levels of PXR has been associated with metabolic syndrome, most commonly liver steatosis and obesity, but the mechanistic rationale is unclear [65]. Here, we predict that increased activation of PXR target genes can cause an increased potential for glucose production from the liver, an important driver of metabolic syndrome.

## Conclusion

The central role of estrogens in mammalian biology is clear. However, the complex nature of this biology, with multiple factors impacting on estradiol kinetics and multiple biological targets effected by the hormone, raises an important challenge with regard to predicting these effects. This is especially pertinent given the increasing concern about environmental exposure to endocrine disrupting compounds, and the epidemiological evidence linking such exposure to chronic conditions. PBPK modelling has traditionally been used to predict drug disposition within individuals and populations. Such models are informed by, and validated through, experimental data, with a lack of correlation highlighting areas for model refinement in a truly iterative process [19, 66]. One advantage of such approaches is that they do not try to model all potential interactions between the drug and the body. Rather, they use a reductionist approach to reproduce the main emergent behaviours of the system. However, this is also a disadvantage as mechanistic detail is lost at the expense of model reduction. To balance these pros and cons, we and others propose the addition of biologically important, mechanistically-detailed hubs within a more general reductionist model [17, 18]. To demonstrate such an approach, we have expanded a classical PBPK model of estradiol [20] in two distinct ways; First, through the addition of a fully deterministic kinetic model of estrogen metabolism within the liver; second, through the addition of a genome scale metabolic network to encompass all metabolic pathways in the liver. We demonstrate that all three models can reproduce experimental data on the plasma kinetics of estradiol following both i.v. bolus and oral exposures. The utility of these two approaches is then examined. Generation of a fully deterministic model for chemical metabolism is time-consuming, as it requires the derivation of robust parameters for all ODEs. This may not be possible for all compounds, as full metabolic (and kinetic) profiling will not have been undertaken. However, when generated, such a model has the potential to underpin/inform the setting of an intrinsic clearance term, predicting the contribution of different enzymes on the final clearance rate. In addition, the extra mechanistic information also has the potential to allow examination of specific scenarios, such as the impact of genetic variation. In contrast to the time required to develop a mechanism-based deterministic model, GSMNs are now available ‘off-the-shelf’. In this case, the challenge here has been that analysis of GSMNs has traditionally relied upon the assumption of a steady-state, meaning that the adaption of metabolic systems to chemical exposure cannot be robustly modelled. Here, we use our QSSPN approach to integrate a GSMN with both a PBPK model and an ODE-based model of PXR-mediated gene expression. We demonstrate how these models can reproduce a complex biological phenomenon, and one with important clinical implications: drug-drug interactions. The central advantage of such an approach is that the DDI emerges from the network connectivity captured by the GSMN, rather than having to be described explicitly. Essentially, the induction of PXR-target genes by phenytoin causes a change in the flux bounds for a range of reactions within the GSMN. As some of these reactions are related to estradiol, then its metabolism may be impacted, which would lead to the emergence of the DDI. An advantage of this approach is that the alteration in bounds leads to the possibility of a DDI, but does not necessarily determine that it will occur. If, for example, estradiol metabolism was not limited by the bounds set in the original GSMN then the induction would be biologically silent as there was already sufficient capacity in the system to meet the biological demand.

Finally, we demonstrate how this approach can be used to explore the implications of chronic drug use. Adaptation of metabolism to chemical exposure is a well described phenomenon, and one that underpins the concept of homeostasis. However, such adaptation often comes at a biological cost, with some areas of metabolism being deprioritised to meet the immediate metabolic demand. If exposure to the drug is acute this may have a limited impact, but upon chronic exposure this alteration in metabolic landscape may have significant consequences. For example, some of the adverse health effects associated with chronic drug exposure are almost certainly due to such metabolic reprogramming [64, 65].

We believe that the use of these extended modelling approaches will allow more precise mechanistic questions to be addressed, helping to interpret of complex biological datasets. This will add significant biological insight to understanding drug actions on the body, and the factors that may influence the emergent biological effect [67].

## Additional files

Additional file 1: Full description of PBPK, PBPK-LiverODE and PBPK-GSMN models, including overall structure, reaction parameters, balance equations, global quantities and initial conditions. In addition, further validation and sensitivity analysis of model behaviours are included, as well as further details of the anthropometric data used for the virtual population. (DOCX 1617 kb)

Additional file 2: Sample steady-state flux distributions within the GSMN of the PBPK-GSMN model under both basal (i.e. no phenytoin) and 80mM phenytoin exposure scenarios. (XLSX 376 kb)

## Abbreviations

AUC: Area under the concentration-time curve;
*CLeh*: *Extrahepatic clearance*
Clint: Intrinsic clearance
Cmax: Maximum concentration
DDI: Drug-drug interaction;
ER: Estrogen receptor
GSMN: Genome-scale metabolic network
*i.v.*: Intravenous
ODE: Ordinary differential equation
*p.o.*: Per os (oral)
PBPK: Physiologically-based pharmacokinetics
PN: Petri net
PXR: Pregnane X-receptor
SHBG: Steroid hormone binding globulin
Tmax: Time to maximum concentration
