# Supplementary material for: Linking physiologically-based pharmacokinetic and genome-scale metabolic networks to understand estradiol biology
Source: BMC Syst Biol. 2017 Dec 15;11:141. doi: 10.1186/s12918-017-0520-3 (PMC5732473; doi:10.1186/s12918-017-0520-3)
Supplement: Supplementary file 1 — Full description of PBPK, PBPK-LiverODE and PBPK-GSMN models, including overall structure, reaction parameters, balance equations, global quantities and initial conditions. In addition, further validation and sensitivity analysis of model behaviours are included, as well as further details of the anthropometric data used for the virtual population. (DOCX 1617 kb) [file 12918_2017_520_MOESM1_ESM.docx]

Additional file 1

Linking physiologically-based pharmacokinetic and genome-scale metabolic networks to understand estradiol biology

Joanna H. Sier, Alfred E. Thumser and Nick J. Plant

TABLE OF CONTENTS

[Physiologically-based pharmacokinetic model for estrogen in women 3](#_Toc472932306)

[Figure S1: Physiologically based Pharmacokinetic model for estradiol in women 3](#_Toc472932307)

[Model Overview 4](#_Toc472932308)

[Figure S2: Scaled sensitivity analysis for PBPK model of estrogen in women. 5](#_Toc472932309)

[Reactions 5](#_Toc472932310)

[Balance Equations 7](#_Toc472932311)

[Global Quantities 9](#_Toc472932312)

[Detailed mechanistic model of estrogen metabolism in the liver 11](#_Toc472932313)

[Model Overview 11](#_Toc472932314)

[Model development 12](#_Toc472932315)

[Table S1: Liquid Chromatography inlet gradients used for detection of estrogens in conditioned medium and cell lysates. 14](#_Toc472932316)

[Figure S4: Mechanistic model of estrogen metabolism in the liver reproduces in vitro data. 15](#_Toc472932317)

[Reactions 15](#_Toc472932318)

[Balance Equations 22](#_Toc472932319)

[Global Quantities 24](#_Toc472932320)

[Initial Conditions 24](#_Toc472932321)

[Quasi steady-state Petri net model of estrogen metabolism 25](#_Toc472932322)

[Figure S5: Quasi steady-state Petri net (QSSPN) model of estrogen metabolism. 25](#_Toc472932323)

[Model Overview 25](#_Toc472932324)

[Figure S6: Simulation results for PBPK-GMSN model of estradiol in women. 27](#_Toc472932325)

[Anthropometric Data 28](#_Toc472932326)

[Overview 28](#_Toc472932327)

[Figure S7: Summary anthropometric statistics for NHANES 2013-14 28](#_Toc472932328)

[References 29](#_Toc472932329)

# Physiologically-based pharmacokinetic model for estrogen in women


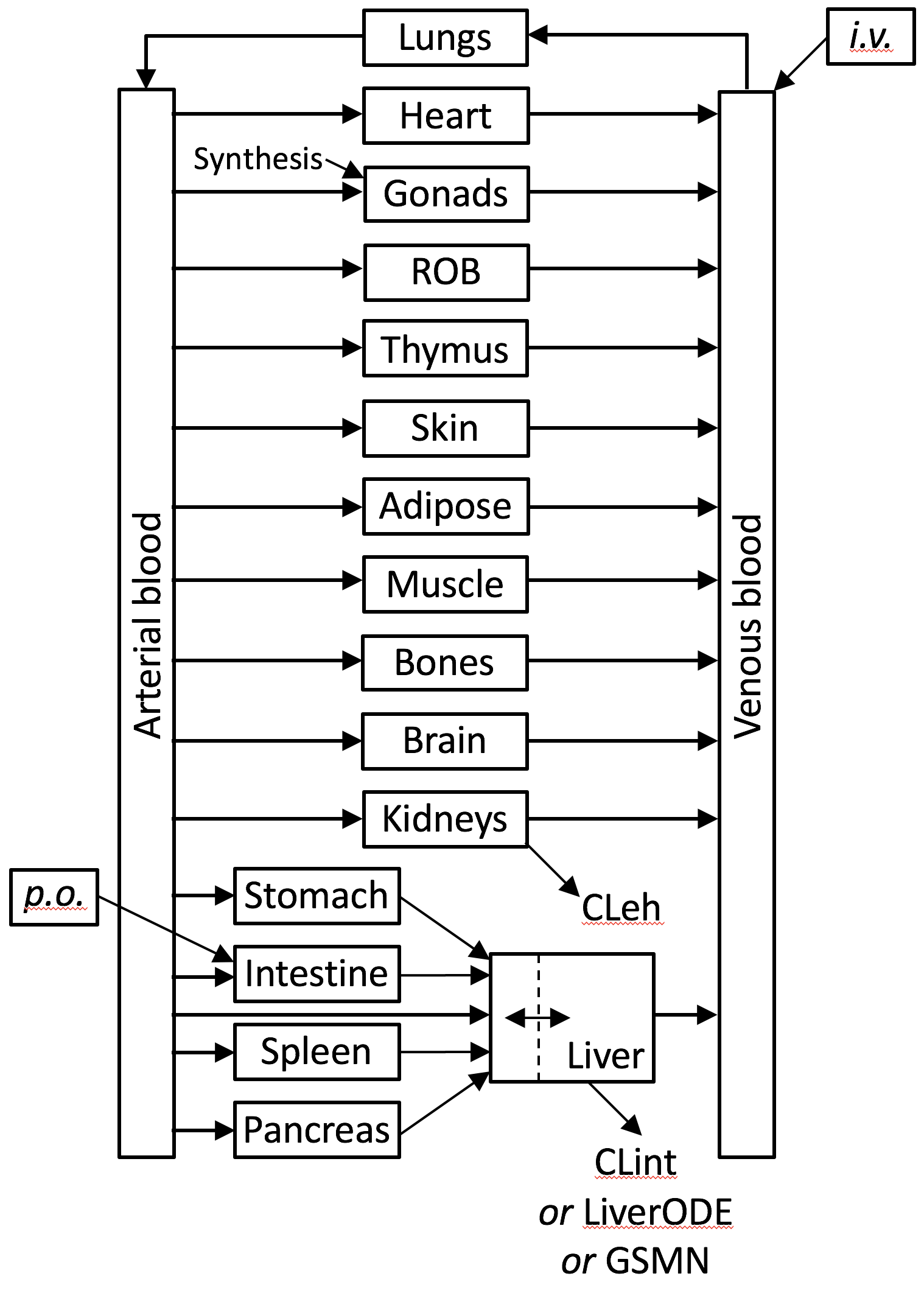


### Figure S1: Physiologically based Pharmacokinetic model for estradiol in women. Distribution of estradiol between venous and arterial blood compartments and 16 tissue compartments is represented. The liver is represented as a permeability-limited tissue, while all other compartments well mixed. Estradiol enters the model through synthesis into the gonads, oral dosing (p.o.) into the intestine, and intravenous dosing (i.v.) into the venous blood. Estradiol is removed from the model through extra-hepatic clearance (CLeh) from the kidney, and intrinsic clearance from the liver. Intrinsic clearance is modelled as either a single ODE (Clint), ODE-based model of liver metabolism (LiverODE), or a genome-scale metabolic network (GSMN) as described in text.

## Model Overview

The PBPK model was created in COPASI v4.14 [1], and was based upon the previously published human female model of Plowchalk and Teeguarden [2]. The model is comprised of venous and arterial blood compartments, plus 16 tissue compartments. With the exception of the liver, all compartments are described as well-mixed, rapid equilibrium compartments. The liver is treated as a permeability-limited compartment with a separate tissue blood compartment [2, 3]. Finally, compartments to represent *i.v.* and oral delivery were added to the model, inputting into the venous blood and intestine compartments, respectively.

As estradiol is an endogenous compound, an estrogen biosynthesis reaction was added using mass action kinetics, mimicking the production of estradiol in the uterus. The rate constant for this reaction was fitted to achieve a steady-state blood concentration of 0.15nM total estradiol, consistent with the published literature [4, 5]. As estradiol in the blood is approximately 98% bound to plasma proteins, this equates to a free plasma concentration of approximately 0.003 nM [6].

Generic physiological parameters were taken from Bosgra [3] and estradiol-specific parameters from Plowchalk and Teeguarden [2],. These were used to populate ordinary differential equations as described by Peters [7].

To examine the impact of parameter robustness on model predictions we performed a sensitivity analysis, with steady-state concentration of plasma estradiol as the measured effect. As shown in figure S2, body weight and estradiol biosynthesis rate were the two most sensitive parameters. Body weight is an important anthropometric value, and used to estimate blood volume though the prediction of the body surface area. As such, it is not surprising that this is a sensitive factor, displaying an inverse relationship to E2{venous}, and in fact reflects an important biological relationship. The biosynthesis rate has an direct relationship with E2{venous}, reflecting the fact that increased input of E2 into the model will produce an increased E2 steady-state level if clearance remains constant. As such, the sensitivity of this parameter may be of more concern as it will have significant impact on basal E2 concentrations, receptor occupancy etc. To mitigate this risk, the biosynthesis rate was fitted to repordice the observed experimental data on basal E2 steady-state concentrations in the blood, and is fixed throughout all simulations. As such, while this parameter has a high potential to impact on simulation results, it is constrained in a manner that will limit its impact on model predictions.

### Figure S2: Scaled sensitivity analysis for PBPK model of estrogen in women.

## Reactions

| **Reactions** | | **Parameters** | **Ref** |
| --- | --- | --- | --- |
| *v1* | Adipose: E2 exit to venous blood  $Q_{adipose}* \frac{{E2}_{adipose(blood)}}{\frac{Kp}{BP}}$ [nM/h] | Kp = 1  BP = 1 | [2, 8] |
| *v2* | Adipose: E2 input from arterial blood  ${E2}_{artery}*Q_{adipose}$ [nM/h] |  |  |
| *v4* | Artery: E2 binding to Albumin  ${E2}_{artery}*{Albumin}_{artery}*k1-{E2.Albumin}_{artery}*k2$ [nM/h] | k1 = 3600 h^-1^  k2 = 6.12e+7 h^-1^ | [9] |
| *v5* | Artery: E2 binding to SHBG  ${E2}_{artery}*{SHBG}_{artery}*k1-{E2.SHBG}_{artery}*k2$ [nM/h] | k1 = 3600 h^-1^  k2 = 5400 h^-1^ | [9] |
| *v6* | Bone: E2 exit to venous blood  $Q_{bone}* \frac{{E2}_{bone}}{\frac{Kp}{BP}}$ [nM/h] | Kp = 1  BP = 1 | [2, 8] |
| *v7* | Bone: E2 input from arterial blood  ${E2}_{artery}*Q_{bone}$ [nM/h] |  |  |
| *v8* | Brain: E2 exit to venous blood  $Q_{brain}* \frac{{E2}_{brain(blood)}}{\frac{Kp}{BP}}$ [nM/h] | Kp = 1  BP = 1 | [2, 8] |
| *v9* | Brain: E2 input from arterial blood  ${E2}_{artery}*Q_{brain}$ [nM/h] |  |  |
| *v10* | Clearance: Extra-hepatic  ${E2}_{kidney}*k1$ [nM/h] | k1 = 5 h^-1^ | [2] |
| *v11* | Clearance: Intrinsic  ${E2}_{liver(cells)}*k1$ [nM/h] | k1 = $(0.018466*liver weight)$ h^-1^ | * |
| *v12* | Dose: IV_bolus  ${E2}_{Dose\_IV}*k1$ [nM/h] | K1 = 250 h^-1^ |  |
| *V13* | Dose: IV_infusion  $k1$ [nM/h] | K1 = as required | [2] |
| *V14* | Dose: oral  ${E2}_{Dose\_oral}*k1$ [nM/h] | K1 = 0.025 h^-1^ |  |
| *v15* | Gonads: Exit to venous  $Q_{gonads}* \frac{{E2}_{gonads(blood)}}{\frac{Kp}{BP}}$ [nM/h] | Kp = 1  BP = 1 | [2, 8] |
| *v16* | Gonads: E2 input from arterial blood  ${E2}_{artery}*Q_{gonads}$ [nM/h] |  |  |
| *v17* | Gonads: E2 biosynthesis  $Biosynthesis*k1$ [nM/h] | k1 = 5 h^-1^ |  |
| *v18* | Gonads: E2 binding to ER  ${E2}_{gonads(cells)}*{ER}_{gonads(cells)}*k1-{E2.ER}_{liver(cells)}*k2$ [nM/h] | k1 = 1000 h^-1^  k2 = 100 h^-1^ | [2, 10] |
| *v19* | Gonads: Permeability-limited access  ${E2}_{gonads(blood)}*k1-{E2}_{gonads\left( cells \right)}*k2$ [nM/h] | k1 = 1000 h^-1^  k2 = 100 h^-1^ | [2, 10] |
| *V20* | Heart: E2 exit to venous blood  $Q_{heart}* \frac{{E2}_{heart}}{\frac{Kp}{BP}}$ [nM/h] | Kp = 1  BP = 1 | [2, 8] |
| *V21* | Heart: E2 input from arterial blood  ${E2}_{artery}*Q_{heart}$ [nM/h] |  |  |
| *V22* | Intestine: E2 input from arterial blood  ${E2}_{artery}*Q_{intestine}$ [nM/h] |  |  |
| *v23* | Kidney: E2 exit to venous blood  $Q_{kidney}* \frac{{E2}_{kidney}}{\frac{Kp}{BP}}$ [nM/h] | Kp = 1  BP = 1 | [2, 8] |
| *v24* | Kidney: E2 input from arterial blood  ${E2}_{artery}*Q_{kidney}$ [nM/h] |  |  |
| *v25* | Liver: E2 binding to ER  ${E2}_{liver(cells)}*{ER}_{liver(cells)}*k1-{E2.ER}_{liver(cells)}*k2$ [nM/h] | k1 = 3600 h^-1^  k2 = 900 h^-1^ | [2] |
| *V26* | Liver: E2 exit to venous blood  $Q_{liver(venous)}* \frac{{E2}_{liver(blood)}}{\frac{Kp}{BP}}$ [nM/h] | Kp = 1  BP = 1 | [2, 8] |
| *v27* | Liver: E2 input from arterial blood  ${E2}_{artery}*Q_{liver(artery)}$ [nM/h] |  |  |
| *v28* | Liver: E2 input from intestine  ${E2}_{intestine}*Q_{intestine}$ [nM/h] |  |  |
| *v29* | Liver: E2 input from spleen  ${E2}_{spleen}*Q_{spleen}$ [nM/h] |  |  |
| *v30* | Liver: E2 input from stomach  ${E2}_{stomach}*Q_{stomach}$ [nM/h] |  |  |
| *v31* | Liver: E2 input from pancreas  ${E2}_{pancreas}*Q_{pancreas}$ [nM/h] |  |  |
| *v32* | Liver: Permeability-limited access  ${E2}_{liver(blood)}*k1-{E2}_{liver\left( cells \right)}*k2$ [nM/h] | k1 = 1000 h^-1^  k2 = 277.8 h^-1^ | [2] |
| *v33* | Lung: E2 exit to arterial blood  $Q_{lung}* \frac{{E2}_{lung}}{\frac{Kp}{BP}}$ [nM/h] | Kp = 1  BP = 1 | [2, 8] |
| *v34* | Lung E2 input from venous blood  ${E2}_{venous}*Q_{lung}$ [nM/h] |  |  |
| *v35* | Muscle: E2 exit to venous blood  $Q_{muscle}* \frac{{E2}_{muscle(blood)}}{\frac{Kp}{BP}}$ [nM/h] | Kp = 1  BP = 1 | [2, 8] |
| *v36* | Muscle: E2 input from arterial blood  ${E2}_{artery}*Q_{muscle}$ [nM/h] |  |  |
| *v37* | E2 oral elimination  ${E2}_{venous}*Q_{lung}$ [nM/h] | k1 = 0.01 h^-1^ | [2] |
| *v38* | Pancreas: E2 input from arterial blood  ${E2}_{Dose\_oral}*k1$ [nM/h] |  |  |
| *v39* | Rest-of-body (ROB): E2 exit to venous blood  $Q_{ROB}* \frac{{E2}_{ROB}}{\frac{Kp}{BP}}$ [nM/h] | Kp = 1  BP = 1 | [2, 8] |
| *v40* | Rest-of-body (ROB): E2 input from arterial blood  ${E2}_{artery}*Q_{ROB}$ [nM/h] |  |  |
| *V41* | Skin: E2 exit to venous blood  $Q_{skin}* \frac{{E2}_{skin(blood)}}{\frac{Kp}{BP}}$ [nM/h] | Kp = 1  BP = 1 | [2, 8] |
| *v42* | Skin: E2 input from arterial blood  ${E2}_{artery}*Q_{skin}$ [nM/h] |  |  |
| *v43* | Spleen: E2 input from arterial blood  ${E2}_{artery}*Q_{spleen}$ [nM/h] |  |  |
| *v44* | Stomach: E2 input from arterial blood  ${E2}_{artery}*Q_{stomach}$ [nM/h] |  |  |
| *v45* | Thymus: E2 exit to venous blood  $Q_{thymus}* \frac{{E2}_{thymus}}{\frac{Kp}{BP}}$ [nM/h] | Kp = 1  BP = 1 | [2, 8] |
| *v46* | Thymus: E2 input from arterial blood  ${E2}_{artery}*Q_{thymus}$ [nM/h] |  |  |
| *v47* | Venous: E2 binding to Albumin  ${E2}_{venous}*{Albumin}_{venous}*k1-{E2.Albumin}_{venous}*k2$ [nM/h] | k1 = 3600 h^-1^  k2 = 6.12e+7 h^-1^ | [9] |
| *v48* | Venous: E2 binding to SHBG  ${E2}_{venous}*{SHBG}_{venous}*k1-{E2.SHBG}_{venous}*k2$ [nM/h] | k1 = 3600 h^-1^  k2 = 5400 h^-1^ | [9] |

## Balance Equations

| $\frac{\partial E2(venous)}{\partial t}$ | *v1 + v6 + v8 + v12 + v13 + v15 + v20 + v23 + v26 + v35 + v39 + v41 + v45 – v34 – v47 – v48* [nM/h] |
| --- | --- |
| $\frac{\partial SHBG(venous)}{\partial t}$ | *–v48* [nM/h] |
| $\frac{\partial E2.SHBG(venous)}{\partial t}$ | *v48* [nM/h] |
| $\frac{\partial Albumin(venous)}{\partial t}$ | *–v47* [nM/h] |
| $\frac{\partial E2.Albumin(venous)}{\partial t}$ | *v47* [nM/h] |
| $\frac{\partial E2(arterial)}{\partial t}$ | *v33 – v2 – v4 – v5 – v7 – v9 – v16 – v21 – v22 – v24 – v27 – v36 – v38 – v40 – v42 – v43 – v44 – v46* [nM/h] |
| $\frac{\partial SHBG(arterial)}{\partial t}$ | *–v5* [nM/h] |
| $\frac{\partial E2.SHBG(arterial)}{\partial t}$ | *v5* [nM/h] |
| $\frac{\partial Albumin(arterial)}{\partial t}$ | *–v4* [nM/h] |
| $\frac{\partial E2.Albumin(arterial)}{\partial t}$ | *v4* [nM/h] |
| $\frac{\partial E2(Adipose)}{\partial t}$ | *v2 – v1* [nM/h] |
| $\frac{\partial E2(Bone)}{\partial t}$ | *v7 – v6* [nM/h] |
| $\frac{\partial E2(Brain)}{\partial t}$ | *v9 – v8* [nM/h] |
| $\frac{\partial E2(Gonads\_blood)}{\partial t}$ | *v16 – v15 – v19* [nM/h] |
| $\frac{\partial E2(Gonads\_cells)}{\partial t}$ | v19 – v18 |
| $\frac{\partial ER(Gonads\_cells)}{\partial t}$ | -v18 |
| $\frac{\partial E2.ER(Gonads\_cells)}{\partial t}$ | v18 |
| $\frac{\partial E2(Heart)}{\partial t}$ | *V21 – v20* [nM/h] |
| $\frac{\partial E2(Intestine)}{\partial t}$ | *V22 + v14 – v28 – v37* [nM/h] |
| $\frac{\partial E2(Kidney)}{\partial t}$ | *v24 – v23 – v10* [nM/h] |
| $\frac{\partial E2(Liver\_blood)}{\partial t}$ | *v27 + v28 + v29 + v30- + v31 – v26- v32* [nM/h] |
| $\frac{\partial E2(Liver\_cells)}{\partial t}$ | *v32 – v25 – v11* [nM/h] |
| $\frac{\partial ER(Liver\_cells)}{\partial t}$ | *-v25* [nM/h] |
| $\frac{\partial E2.ER(Liver\_cells)}{\partial t}$ | *v25* [nM/h] |
| $\frac{\partial E2(Lung)}{\partial t}$ | *v34 – v33* [nM/h] |
| $\frac{\partial E2(Muscle)}{\partial t}$ | *v36 - v35* [nM/h] |
| $\frac{\partial E2(Pancreas)}{\partial t}$ | *v38 – v31* [nM/h] |
| $\frac{\partial E2(Rest.of.Body)}{\partial t}$ | *V40 – v39* [nM/h] |
| $\frac{\partial E2(Skin)}{\partial t}$ | *v42 – v41* [nM/h] |
| $\frac{\partial E2(Spleen)}{\partial t}$ | *V43 – v29* [nM/h] |
| $\frac{\partial E2(Stomach)}{\partial t}$ | *v44 – v30* [nM/h] |
| $\frac{\partial E2(Thymus)}{\partial t}$ | *v46 – v45* [nM/h] |

## Global Quantities

| **Parameter** | | **Assignment** | **Ref** |
| --- | --- | --- | --- |
| **Compartments Volumes (L)** | |  |  |
| Adipose | | $\frac{Body weight*\left( \frac{1.2*BMI+0.23*Age-10.8*0-5.4}{100} \right)}{0.916}$ | [11] |
| Plasma_arterial | | $\frac{\left( 2.66*BSA-0.46 \right)*0.33*0.55}{1.025}$ | [3] |
| Plasma_venous | | $\frac{\left( 2.66*BSA-0.46 \right)*0.67*0.55}{1/025}$ | [3] |
| Bone | | $\frac{e^{2.67*\ln\left( Height \right)+0.0689}}{1.99}$ | [3] |
| Brain | | $\frac{0.373*\left\{ \left( 3.68-2.68*e^{\frac{-Age}{0.89}} \right)*\left( e^{\frac{-Age}{629}} \right) \right\}}{1.035}$ | [3] |
| Gonads | | $\frac{\frac{3.3+90*\left( 1-e^{-\left( \frac{Age}{16.8} \right)^{6.7}} \right)}{1000}}{1.03}$ | [3] |
| Heart | | $\frac{e^{2.13*\ln\left( Height \right)-2.502}}{1.03}$ | [3] |
| Intestine | | $\frac{e^{2.47*\ln\left( Height \right)-1.351}}{1.042}$ | [3] |
| Kidney | | $\frac{e^{1.93*\ln\left( Height \right)-2.306}}{1.05}$ | [3] |
| Liver_blood | | $Liver\_cells*0.075$ | [2] |
| Liver_cells | | $\frac{e^{1.98*\ln\left( Height \right)-0.6786}}{1.03}$ | [3] |
| Lung | | $\frac{e^{2.1*\ln\left( Height \right)-2.092}}{1.05}$ | [3] |
| Muscle | | $\frac{Body weight*0.93-\sum volumes of other compartments}{1.041}$ | [3] |
| Pancreas | | $\frac{e^{2.43*\ln\left( Height \right)-3.431}}{1.045}$ | [3] |
| Rest-of-body | | $\frac{e^{1.*\ln\left( Height \right)-0.072}}{1.03}$ | [3] |
| Skin | | $\frac{e^{2.63*\ln\left( Height \right)-1.93}}{1.5}$ | [3] |
| Spleen | | $\frac{e^{2.16*\ln\left( Height \right)-3.123}}{1.054}$ | [3] |
| Stomach | | $\frac{e^{2.45*\ln\left( Height \right)-3.266}}{1.44}$ | [3] |
| Thymus | | $\frac{\frac{14*\left\{ \left( 7.1-6.1*e^{\frac{-Age}{11.9}} \right)*\left( 0.14+0.86*e^{\frac{-Age}{10.3}} \right) \right\}}{1000}}{1.03}$ | [3] |
| **Parameter** | | **Assignment** | **Ref** |
| **Organ Blood Flow (L.h^-1^)** | |  |  |
| Q1 | Adipose | $Adipose_{cells}volume*1.4*0.916*0.55$ | [3] |
| Q2 | Bone | $Bone volume*1.8*1.99*0.55$ | [3] |
| Q3 | Brain | $Brain_{cells}volume*32.7*1.035*0.55$ | [3] |
| Q4 | Gonads | $Gonads_{cells}volume*0.8*1.03*0.55$ | [3] |
| Q5 | Heart | $Heart volume*70.8*1.03*0.55$ | [3] |
| Q6 | Intestine | $Intestine volume*59.0*1.042*0.55$ | [3] |
| Q7 | Kidney | $Kidney volume*$215*1.05*0.55 | [3] |
| Q8 | Liver (arterial) | $Liver_{cells}volume*16.4*1.03*0.55$ | [3] |
| Q9 | Liver (venous) | $liver\left( arterial \right)+intestine+pancreas+spleen+stomach$ | [3] |
| Q10 | Lung | $Cardiac output$ | [3] |
| Q11 | Muscle | $Muscle volume*2.4*1.041*0.55$ | [3] |
| Q12 | Pancreas | $Pancreas volume*29.5*1.045*0.55$ | [3] |
| Q13 | Rest-of-Body | $ROB volume*18.8*1.03*0.55$ | [3] |
| Q14 | Skin | $Skin_{cells}volume*7.7*1.5*0.55$ | [3] |
| Q15 | Spleen | $Spleen volume*81.5*1.054*0.55$ | [3] |
| Q16 | Stomach | $Stomach volume*25.3*1.044*0.55$ | [3] |
| Q17 | Thymus | $Thymus volume*18.5*1.03*0.55$ | [3] |
| CO | Cardiac Output | $\sum_{\begin{aligned} i=n \\ i\neq8,10 \end{aligned}}^{17} Qi$ |  |
| **Parameter** | | **Assignment** | **Ref** |
| **Other Measurements** | |  |  |
| Body Surface Area (BSA) | | $0.007184*{Body weight}^{0.425}* {Height}^{0.725}$ | [3] |
| Body Mass Index (BMI) | | $\frac{Body weight}{{Height}^{2}}$ |  |
| Liver weight (g) | | $Liver_{cells}*1000*1.03$ |  |

# Detailed mechanistic model of estrogen metabolism in the liver


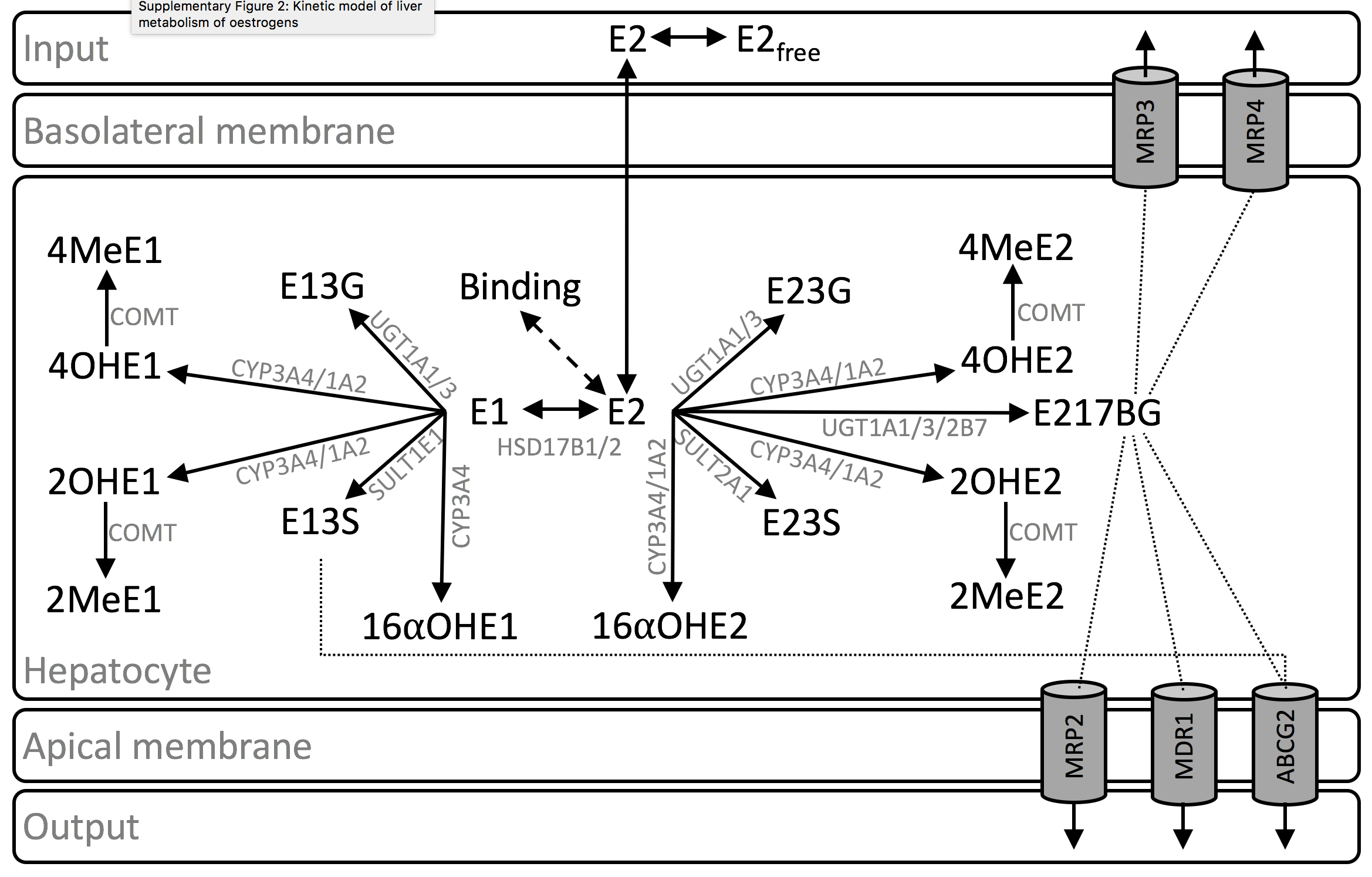


Figure S3: Mechanisitic model of estrogen metabolism in the liver. Compartments are indicated as boxes, metabolic conversions as solid lines, binding reactions as dashed lines and transport reactions as dotted lines. Enyzmes responsible for metabolic conversions/transport reactions are indicated. E2=estradiol and E1=estrone. For both E1 and E2, metabolites are identified as 3G=3-glucoronide; 3S=3-sulphate; 2OH, 4OH and 16αOH=hydroxylation at 2, 4 and 16α positions; 2Me and 4Me=methoxylation at 2 and 4 positions.

## Model Overview

The liver model for estradiol metabolism was created in COPASI v4.14 [1] and comprises five compartments: an input compartment representing either medium or blood; an output compartment representing medium or bile; and three compartments representing parts of the hepatocyte, namely apical membrane space, basolateral membrane space, and cell interior. Within the input compartment, estradiol is present as E2 and E2free to represent diffusion-limited access of estradiol to cells. E2free represents the bulk of estradiol in the input compartment, while E2 represents estradiol immediately bordering cells that can gain access to the cell membrane and enter cells. This diffusion-limited access is simulated through first order kinetics, with a rate constant based upon previously used estimates for chemical diffusion through medium [12]. Movement between compartments is through either passive diffusion or active transport. Within the hepatocyte, estradiol is subject to sequestration through binding. Both specific (*i.e.* binding to the estrogen receptor) and non-specific binding are included Estradiol undergoes a number of metabolic conversions, indicated by arrows with the responsible enzyme(s) named. Briefly, estradiol is intraconverted with estrone through the actions of 17β‑hydroxysteroid dehydrogenase 1 and 2 [13, 14]. Both estradiol and estrone are metabolised via Phase I and Phase II: CYP1A1 and CYP3A4 during Phase I metabolism [15, 16] and SULT1E1, SULT2A1, UGT1A1, UGT1A3 and UGT2B7 during Phase II metabolism [17]. In addition, the catecholamine metabolites of E1 and E2 CYP-mediated hydroxylation are potent mutagens, are readily deactivated through the action of catechol-O-methyltransferase (COMT) [18].

The kinetic parameters for enzymatic and active transport reactions (Km and Kcat) were either obtained directly from the published literature or inferred from experimental data. Protein abundances were retrieved from the Model Organism Protein Expression Database (MOPED) [19]. Calculation of passive diffusion rate constants was through the method of van de Waterbeemd [20], with logP values retrieved from the Human Metabolome Database (HMBD) [6]. Where experimental logP values were not available, the ALOGPS prediction was used, which has been extensively validated, with root mean square error <0.35 across approximately 13,000 predictions [21].

## Model development

The mechanistic model of estradiol metabolism in the liver was based upon the known fates of estradiol in the liver as described by current literature: these include Phase I and II metabolism, intracellular binding (both specific and non-specific), and membrane transport (passive and active). For each protein species, concentrations were taken from the protein abundance database MOPED, while kinetic parameters were obtained either from the published literature or inferred from experimental data, as indicated in the supplementary information. Steady-state concentrations of all species within the model were predicted as follows: first, the basal levels of estradiol in primary human hepatocytes was determined as approximately 15nM by UPLC-MS (see below for method). Estradiol from the medium is expected to contribute little to this value, as naïve mediums have been reported to possess low levels of estrogens (0.4nM) [22]. Second, a biosynthesis term was introduced in to the model to reflect endogenous production of estrogens in hepatocytes. Third, all terminal species in the network (i.e. medium and bile compartment species) were fixed with the exception of estradiol and estrone in the medium. Fourth, the biosynthesis rate constant was varied until steady-state concentrations of estradiol were consistent with the experimental data.

## Measurement of estradiol metabolism in primary human hepatocytes

Due to the mechanistic nature of the model architecture, and the use of experimentally derived protein abundance and enzyme kinetic parameters, the model should accurately reproduce the metabolism and disposition of estradiol within a healthy human liver, or more precisely the hepatocyte component of the liver, without the requirement for any data fitting. To examine this, we exposed primary human hepatocytes to 1000 nM estradiol *in vitro*, and measured the levels of estradiol in the medium and cell lysate over 8 hours by UPLC-MS.

Female primary human hepatocytes were a kind gift from Dr Katherine Fenner (Pfizer, Sandwich, UK). Lebovitz L-15 medium with L-glutamine and amino acids, plus 100x non-essential amino acids were purchased from Invitrogen (Paisley, UK). Estradiol, estrone, sodium glycolate, 2-(N-morpholino)ethanesulfonic acid and poly-L-lysine were purchased from Sigma-Aldrich (Poole, UK). LC-MS measurement of estradiol and estrone was undertaken using a Waters H-class UPLC system, coupled with a Xevo-TQS, Acquity BEH C8 1.7um, 2.1 x 5mm van guard pre-column and an Acquity BEH C8 1.7 μm 2.1 x 100 mm column (Waters, Elstree, UK). Optima grade methanol and water were purchased from Fisher Scientific (Loughborough, UK), and amber samples vial, inserts and PTFE/Silicon caps were purchased from Chromacol (VWR, Lutterworth, UK). All other reagents were of cell culture or molecular grade, as appropriate, and purchased from Sigma-Aldrich.

Estradiol was prepared at 1 mg/ml with 0.1 % DMSO (v/v) in optima grade methanol, and then diluted to 1 µg/ml estradiol with 2 mM sodium glycocholate in optima grade methanol to enhance solubility. Samples were dried under nitrogen and re-suspended in HBSS buffer supplemented with 10 mM 2-(N-morpholino)ethanesulfonic acid. Primary human hepatocytes were seeded at 3.3 x 106 cells/cm2 in Lebovitz L-15 medium with L-glutamine and amino acids. Plates were incubated for 24 hours to allow cell adherence, and then cells exposed to 1000nM estradiol in serum-free medium. Samples of conditioned medium and cell lysate were taken at 0, 15, 30, 45, 60, 90, 120, 150, 180, 210, 240 minutes and then hourly until 8 hours.

Conditioned medium samples were prepared for UPLC-MS analysis as follows: conditioned medium was vortexed for 30 seconds, left for 30 minutes at room temperature, and vortexed again. Acetonitrile (1:1 v/v) was added, the sample vortexed for 30 seconds and left at room temperature for 30 minutes. Samples were then vortexed one final time and centrifuged at 10,000 xg for 15 minutes. Cell lysates were prepared for UPLC-MS analysis as follows: cells were washed in ice-cold (4˚C) water and then harvested by scraping. The cell suspension was pipetted up and down vigorously and an equal volume of ice-cold acetonitrile (v/v) added. Samples were vortexed for 30 seconds, left on ice for 30 minutes, and then vortexed for a further 30 seconds. Finally, samples were centrifuged at 10,000 xg at 4˚C for 15 minutes.

For UPLC-MS detection of estradiol in conditioned medium the methodology of Guo et al. [23] was used, with modifications. The separation of metabolites was carried out under reverse phase conditions using Fisher optima grade water as mobile phase A and optima grade methanol as mobile phase B. Supplementary table S1 shows the gradient and flow rates used. Capillary, cone and collision voltages were set to 1.6 KV, 30 V and 32 V, respectively. The parent ion for estradiol was detected at 269.2 m/z, while daughter ions were detected at 145.8 m/z and 161.1 m/z.

| **Time** | **Solvent A (%)** | **Solvent B (%)** |
| --- | --- | --- |
| 0 | 98 | 2 |
| 1 | 98 | 2 |
| 2 | 55 | 45 |
| 8 | 55 | 45 |
| 9 | 10 | 90 |
| 10 | 10 | 90 |
| 15 | 98 | 2 |
| 17 | 98 | 2 |

Table S1: Liquid Chromatography inlet gradients used for detection of estrogens in conditioned medium and cell lysates. Solvent A = Optima grade water and solvent B = optima grade methanol. Flow rate was 0.4 mL/min

Estradiol within the medium exhibited a time-dependent decrease in concentration, consistent with first order kinetics (figure S4a). We simulated the response of the metabolic model to 1000 nM estradiol, and compared this to experimental data (Figure 4a). The simulation predicts that estradiol rapidly enters the cell, with intracellular levels peaking within the first ten minutes before decreasing over the next 8 hours. Initial simulations demonstrated a non-linear decrease over time, but significantly underpredicted the level of estrogen remaining in the medium, suggesting that the rate of clearance was too high. It is well established that primary hepatocytes lose metabolic capacity over time in culture, and that this loss is not even across all enzyme classes [24, 25]. To compensate for this, ‘a protein expression factor’ is incorporated into the model, allowing the expression of all proteins in the model to be scaled appropriately. When a protein expression factor of 0.1 was used the predicted estradiol concentration over time was contained within the 95% confidence intervals for the experimental data at all time points. Given the use of three replicates for the experimental data, this comparison has a power of 77%.

To examine the impact of parameter robustness on model predictions we performed a sensitivity analysis, with steady-state concentration of medium estradiol as the measured effect. As shown in figure S4b, the protein expression factor and biosynthesis rate were the two most sensitive parameters. While these are fitted to meet the observed experimental data, they are held constant through all simulations, and hence this sensitivity is not a significant factor. As expected, Kcat and Km values for a number of metabolic enzymes were also shown to have an impact on predicted medium estradiol concentrations. Derivation of robust Km paramaters for these reactions is relatively straightforward, with several publications supporting a predicted affinity within a two-fold margin of error. In comparison, the calculation of Kcat is more complex, as it relies on two factors: accurate estimation of protein levels in hepatocytes; and, estimation of the protein concentration in the system(s) used for assessment of kinetic data. While the first factor can be derived from databases such as MOPED [19], the second factor is more complex to derive as the required information is rarely presented in the literature. As such, estimates of Kcat are more likely to deviate from the actual value by larger amounts. While this should be remembered as a caveat for the model, it should be noted that the overall impact of any errors is minimal as it allows the robust reproduction of the in vitro metabolism of estradiol. However, in situation where the level of individual proteins and/or their activity is examined (e.g. prediction of genetic variation), this uncertainty may be more important.

Figure S4: Mechanistic model of estrogen metabolism in the liver reproduces in vitro data. (A) Primary human hepatocytes were exposed to 1000nM estradiol, and then the concentration of medium estradiol measured over eight hours using LC-MS. Symbols represent mean concentration from three independent biological replicates, with dashed lines representing the 95% confidence interval of the regression curve. Solid lines represent simulated data. (B) Scaled sensitivity for medium estradiol

## Reactions

| **Reactions** | | **Parameters** | **Refs** |
| --- | --- | --- | --- |
| *v1* | Bile: E13S Clearance  ${E13S}_{bile}*k1$ [nM/h] | k1 = 0 |  |
| *v2* | Bile: E217BG Clearance  ${E217BG}_{bile}*k1$ [nM/h] | k1 = 0 |  |
| *v3* | Hep: 2OHE1 -> 2MeE1; COMT  $\frac{2OHE1*kcat*COMT}{2OHE1+Km}$ [nM/h] | kcat = 198 h^-1^  Km = 7400 nM | [18, 26, 27] |
| *v4* | Hep: 2OHE2 -> 2MeE2; COMT  $\frac{2OHE2*kcat*COMT}{2OHE2+Km}$ [nM/h] | kcat = 408 h^-1^  Km = 108000 nM | [18, 26, 27] |
| *v5* | Hep: 4OHE1 -> 4MeE1; COMT  $\frac{4OHE1*kcat*COMT}{4OHE1+Km}$ [nM/h] | kcat = 402 h^-1^  Km = 53000 | [18, 26, 27] |
| *v6* | Hep: 4OHE2 -> 4MeE2; COMT  $\frac{4OHE2*kcat*COMT}{4OHE2+Km}$ [nM/h] | kcat = 204 h^-1^  Km = 24000 nM | [18, 26, 27] |
| *v7* | Hep: E1 -> 16αOHE1; CYP3A4  $\frac{E1*kcat*CYP3A4}{E1+Km}$ [nM/h] | kcat = 30 h^-1^  Km = 64000 nM | [28, 29] |
| *v8* | Hep: E1 -> 2OHE1; CYP1A2  $\frac{E1*kcat*CYP1A2}{E1+Km}$ [nM/h] | kcat = 552 h^-1^  Km = 1900 nM | [28, 29] |
| *v9* | Hep: E1 -> 2OHE1; CYP3A4  $\frac{E1*kcat*CYP3A4}{E1+Km}$ [nM/h] | kcat = 42 h^-1^  Km = 102000 nM | [28, 29] |
| *v10* | Hep: E1 -> 4OHE1; CYP1A2  $\frac{E1*kcat*CYP1A2}{E1+Km}$ [nM/h] | kcat = 120 h^-1^  Km = 17000 nM | [28, 29] |
| *v11* | Hep: E1 -> 4OHE1; CYP3A4  $\frac{E1*kcat*CYP3A4}{E1+Km}$ [nM/h] | kcat = 30 h^-1^  Km = 78000 nM | [28, 29] |
| *v12* | Hep: E1 -> E13G; UGT1A1  $\frac{E1*kcat*UGT1A1}{E2+Km}$ [nM/h] | kcat = 15.2 h^-1^  Km = 38000 nM | [30] |
| *v13* | Hep: E1 -> E13G; UGT1A3  $\frac{E1*kcat*UGT1A3}{E2+Km}$ [nM/h] | kcat = 186.6 h^-1^  Km = 77000 nM | [30] |
| *v14* | Hep: E1 -> E13S; SULT1E1  $\frac{E1*kcat*SULT1E1}{E1+Km}$ [nM/h] | kcat = 8.46e+6 h^-1^  km = 6 nM | [31] |
| *v15* | Hep: E1 -> E2; HSD17B1  $\frac{E1*kcat*HSD17B1}{E2+Km}$ [nM/h] | kcat = 5400 h^-1^  Km = 70000 nM | [32] |
| *v16* | Hep: E2 -> 16αOHE2; CYP1A2  $\frac{E2*kcat*CYP1A2}{E2+Km}$ [nM/h] | kcat = 42 h^-1^  Km = 58000 nM | [29] |
| *v17* | Hep: E2 -> 16αOHE2; CYP3A4  $\frac{E2*kcat*CYP3A4}{E2+Km}$ [nM/h] | kcat = 24 h^-1^  Km = 75000 nM | [29] |
| *v18* | Hep: E2 -> 2OHE2; CYP1A2  $\frac{E2*kcat*CYP1A2}{E2+Km}$ [nM/h] | kcat = 660 h^-1^  Km = 20000 nM | [15, 29, 33] |
| *v19* | Hep: E2 -> 2OHE2; CYP3A4  $\frac{E2*kcat*CYP3A4}{E2+Km}$ [nM/h] | kcat = 48 h^-1^  Km = 54000 nM | [29, 33-35] |
| *v20* | Hep: E2 -> 4OHE2; CYP1A2  $\frac{E2*kcat*CYP1A2}{E2+Km}$ [nM/h] | kcat = 54 h^-1^  Km = 28000 nM | [29] |
| *v21* | Hep: E2 -> 4OHE2; CYP3A4  $\frac{E2*kcat*CYP3A4}{E2+Km}$ [nM/h] | kcat = 18 h^-1^  Km = 111000 nM | [29] |
| *v22* | Hep: E2 -> E1; HSD17B1  $\frac{E2*kcat*HSD17B1}{E2+Km}$ [nM/h] | kcat = 43.4 h^-1^  Km = 2000 nM | [36] |
| *v23* | Hep: E2 -> E1; HSD17B2  $\frac{E2*kcat*HSD17B2}{E2+Km}$ [nM/h] | kcat = 16926 h^-1^  Km = 110000 nM | [37] |
| *v24* | Hep: E2 -> E217βG; UGT1A1  $\frac{E2*kcat*UGT1A1}{E2+Km}$ [nM/h] | kcat = 212.5 h^-1^  Km = 35000 nM | [30] |
| *v25* | Hep: E2 -> E217βG; UGT1A3  $\frac{E2*kcat*UGT1A3}{E2+Km}$ [nM/h] | kcat = 48.5 h^-1^  Km = 35000 nM | [30] |
| *v26* | Hep: E2 -> E217βG; UGT2B7  $\frac{E2*kcat*UGT2B7}{E2+Km}$ [nM/h] | kcat = 102.7 h^-1^  Km = 11000 nM | [30] |
| *v27* | Hep: E2 -> E23G; UGT1A1  $\frac{E2*kcat*UGT1A1}{E2+Km}$ [nM/h] | kcat = 470.6 h^-1^  Km = 23000 nM | [30] |
| *v28* | Hep: E2 -> E23G; UGT1A3  $\frac{E2*kcat*UGT1A3}{E2+Km}$ [nM/h] | kcat = 145.5 h^-1^  Km = 47000 nM | [30] |
| *v29* | Hep: E2 -> E23S; SULT2A1  $\frac{E2*kcat*SULT2A1}{E2+Km}$ [nM/h] | kcat = 93.7 h^-1^  Km = 14 nM | [38, 39] |
| *v30* | Hep: E2 biosynthesis  $Biosynthesis*k1$ [nM/h] | k1 = 0.76 h^-1^ |  |
| *v31* | Hep: E2 binding to ER  $E2*ER*k1-E2.ER*k2$ [nM/h] | k1 = 3600 h^-1^  k2 = 900 h^-1^ | [2] |
| *v32* | Hep: E2 binding to NSB  $E2*NSB*k1-E2.NSB*k2$ [nM/h] | k1 = 0.1 h^-1^  k2 = 0.1 h^-1^ | [2] |
| *v33* | Medium: 16αOHE1 -> degraded  $16\alpha OHE1*k1$ [nM/h] | k1 = 0 |  |
| *v34* | Medium: 16αOHE2 -> degraded  $16\alpha OHE2*k1$ [nM/h] | k1 = 0 |  |
| *v35* | Medium: 2MeE1 -> degraded  $2MeE1*k1$ [nM/h] | k1 = 0 |  |
| *v36* | Medium: 2MeE2 -> degraded  $2MeE2*k1$ [nM/h] | k1 = 0 |  |
| *v37* | Medium: 2OHE1 -> degraded  $2OHE1*k1$ [nM/h] | k1 = 0 |  |
| *v38* | Medium: 2OHE2 -> degraded  $2OHE2*k1$ [nM/h] | k1 = 0 |  |
| *v39* | Medium: 4MeE1 -> degraded  $4MeE1*k1$ [nM/h] | k1 = 0 |  |
| *v40* | Medium: 4MeE2 -> degraded  $4MeE2*k1$ [nM/h] | k1 = 0 |  |
| *v41* | Medium: 4OHE1 -> degraded  $4OHE1*k1$ [nM/h] | k1 = 0 |  |
| *v42* | Medium: 4OHE2 -> degraded  $4OHE2*k1$ [nM/h] | k1 = 0 |  |
| *v43* | Medium: E1 -> degraded  $E1*k1$ [nM/h] | k1 = 0 |  |
| *v44* | Medium: E13G -> degraded  $E13G *k1$ [nM/h] | k1 = 0 |  |
| *v45* | Medium: E13S -> degraded  $E13S *k1$ [nM/h] | k1 = 0 |  |
| *v46* | Medium: E2 -> degraded  $E2*k1$ [nM/h] | k1 = 0 |  |
| *v47* | Medium: E217BG -> degraded  $E217BG*k1$ [nM/h] | k1 = 0 |  |
| *v48* | Medium: E23G -> degraded  $E23G*k1$ [nM/h] | k1 = 0 |  |
| *v49* | Medium: E23S -> degraded  $E23S*k1$ [nM/h] | k1 = 0 |  |
| *v50* | Transport: 16αOHE1 basolat -> hep  $16\alpha OHE1*k1$ [nM/h] | k1 = 7.04 h^-1^ | [6, 20] |
| *v51* | Transport: 16aOHE1 basolat -> medium  $16\alpha OHE1*k1$ [nM/h] | k1 = 7.04 h^-1^ | [6, 20] |
| *v52* | Transport: 16aOHE1 hep -> basolat  $16\alpha OHE1*k1$ [nM/h] | k1 = 3.6 h^-1^ | [6, 20] |
| *v53* | Transport: 16aOHE1 medium -> basolat  $16\alpha OHE1*k1$ [nM/h] | k1 = 3.6 h^-1^ | [6, 20] |
| *v54* | Transport: 16aOHE2 basolat -> hep  $16\alpha OHE2*k1$ [nM/h] | k1 = 6.05 h^-1^ | [6, 20] |
| *v55* | Transport: 16aOHE2 basolat -> medium  $16\alpha OHE2*k1$ [nM/h] | k1 = 6.05 h^-1^ | [6, 20] |
| *v56* | Transport: 16aOHE2 hep -> basolat  $16\alpha OHE2*k1$ [nM/h] | k1 = 3.6 h^-1^ | [6, 20] |
| *v57* | Transport: 16aOHE2 medium -> basolat  $16\alpha OHE2*k1$ [nM/h] | k1 = 3.6 h^-1^ | [6, 20] |
| *v58* | Transport: 2MeE1 basolat -> hep  $2MeE1*k1$ [nM/h] | k1 = 7.1 h^-1^ | [6, 20] |
| *v59* | Transport: 2MeE1 basolat -> medium  $2MeE1*k1$ [nM/h] | k1 = 7.1 h^-1^ | [6, 20] |
| *v60* | Transport: 2MeE1 hep -> basolat  $2MeE1*k1$ [nM/h] | k1 = 3.6 h^-1^ | [6, 20] |
| *v61* | Transport: 2MeE1 medium -> basolat  $2MeE1*k1$ [nM/h] | k1 = 3.6 h^-1^ | [6, 20] |
| *v62* | Transport: 2MeE2 basolat -> hep  $2MeE2*k1$ [nM/h] | k1 = 7.3 h^-1^ | [6, 20] |
| *v63* | Transport: 2MeE2 basolat -> medium  $2MeE2*k1$ [nM/h] | k1 = 7.3 h^-1^ | [6, 20] |
| *v64* | Transport: 2MeE2 hep -> basolat  $2MeE2*k1$ [nM/h] | k1 = 3.6 h^-1^ | [6, 20] |
| *v65* | Transport: 2MeE2 medium -> basolat  $2MeE2*k1$ [nM/h] | k1 = 3.6 h^-1^ | [6, 20] |
| *v66* | Transport: 2OHE1 basolat -> hep  $2OHE1*k1$ [nM/h] | k1 = 6.63 h^-1^ | [6, 20] |
| *v67* | Transport: 2OHE1 basolat -> medium  $2OHE1*k1$ [nM/h] | k1 = 6.63 h^-1^ | [6, 20] |
| *v68* | Transport: 2OHE1 hep -> basolat  $2OHE1*k1$ [nM/h] | k1 = 3.6 h^-1^ | [6, 20] |
| *v69* | Transport: 2OHE1 medium -> basolat  $2OHE1*k1$ [nM/h] | k1 = 3.6 h^-1^ | [6, 20] |
| *v70* | Transport: 2OHE2 basolat -> hep  $2OHE2*k1$ [nM/h] | k1 = 7.9 h^-1^ | [6, 20] |
| *v71* | Transport: 2OHE2 basolat -> medium  $2OHE2*k1$ [nM/h] | k1 = 7.9 h^-1^ | [6, 20] |
| *v72* | Transport: 2OHE2 hep -> basolat  $2OHE2*k1$ [nM/h] | k1 = 3.6 h^-1^ | [6, 20] |
| *v73* | Transport: 2OHE2 medium -> basolat  $2OHE2*k1$ [nM/h] | k1 = 3.6 h^-1^ | [6, 20] |
| *v74* | Transport: 4MeE1 basolat -> hep  $4MeE1*k1$ [nM/h] | k1 = 7.08 h^-1^ | [6, 20] |
| *v75* | Transport: 4MeE1 basolat -> medium  $4MeE1*k1$ [nM/h] | k1 = 7.08 h^-1^ | [6, 20] |
| *v76* | Transport: 4MeE1 hep -> basolat  $4MeE1*k1$ [nM/h] | k1 = 3.6 h^-1^ | [6, 20] |
| *v77* | Transport: 4MeE1 medium -> basolat  $4MeE1*k1$ [nM/h] | k1 = 3.6 h^-1^ | [6, 20] |
| *v78* | Transport: 4MeE2 basolat -> hep  $4MeE2*k1$ [nM/h] | k1 = 7.25 h^-1^ | [6, 20] |
| *v79* | Transport: 4MeE2 basolat -> medium  $4MeE2*k1$ [nM/h] | k1 = 7.25 h^-1^ | [6, 20] |
| *v80* | Transport: 4MeE2 hep -> basolat  $4MeE2*k1$ [nM/h] | k1 = 3.6 h^-1^ | [6, 20] |
| *v81* | Transport: 4MeE2 medium -> basolat  $4MeE2*k1$ [nM/h] | k1 = 3.6 h^-1^ | [6, 20] |
| *v82* | Transport: 4OHE1 basolat -> hep  $4OHE1*k1$ [nM/h] | k1 = 6.6 h^-1^ | [6, 20] |
| *v83* | Transport: 4OHE1 basolat -> medium  $4OHE1*k1$ [nM/h] | k1 = 6.6 h^-1^ | [6, 20] |
| *v84* | Transport: 4OHE1 hep -> basolat  $4OHE1*k1$ [nM/h] | k1 = 3.6 h^-1^ | [6, 20] |
| *v85* | Transport: 4OHE1 medium -> basolat  $4OHE1*k1$ [nM/h] | k1 = 3.6 h^-1^ | [6, 20] |
| *v86* | Transport: 4OHE2 basolat -> hep  $4OHE2*k1$ [nM/h] | k1 = 6.94 h^-1^ | [6, 20] |
| *v87* | Transport: 4OHE2 basolat -> medium  $4OHE2*k1$ [nM/h] | k1 = 6.94 h^-1^ | [6, 20] |
| *v88* | Transport: 4OHE2 hep -> basolat  $4OHE2*k1$ [nM/h] | k1 = 3.6 h^-1^ | [6, 20] |
| *v89* | Transport: 4OHE2 medium -> basolat  $4OHE2*k1$ [nM/h] | k1 = 3.6 h^-1^ | [6, 20] |
| *v90* | Transport: E1 basolat -> hep  $E1*k1$ [nM/h] | k1 = 7.63 h^-1^ | [6, 20] |
| *v91* | Transport: E1 basolat -> medium  $E1*k1$ [nM/h] | k1 = 7.63 h^-1^ | [6, 20] |
| *v92* | Transport: E1 hep -> basolat  $E1*k1$ [nM/h] | k1 = 3.6 h^-1^ | [6, 20] |
| *v93* | Transport: E1 medium -> basolat  $E1*k1$ [nM/h] | k1 = 3.6 h^-1^ | [6, 20] |
| *v94* | Transport: E13G basolat -> hep  $E13G*k1$ [nM/h] | k1 = 5.53 h^-1^ | [6, 20] |
| *v95* | Transport: E13G basolat -> medium  $E13G*k1$ [nM/h] | k1 = 5.53 h^-1^ | [6, 20] |
| *v96* | Transport: E13G hep -> basolat  $E13G*k1$ [nM/h] | k1 = 3.61 h^-1^ | [6, 20] |
| *v97* | Transport: E13G medium -> basolat  $E13G*k1$ [nM/h] | k1 = 3.61 h^-1^ | [6, 20] |
| *v98* | Transport: E13S apical -> bile  $E13S*k1$ [nM/h] | k1 = 5.04 h^-1^ | [6, 20] |
| *v99* | Transport: E13S apical -> hep  $E13S*k1$ [nM/h] | k1 = 5.04 h^-1^ | [6, 20] |
| *v100* | Transport: E13S basolat -> hep  $E13S*k1$ [nM/h] | k1 = 5.04 h^-1^ | [6, 20] |
| *v101* | Transport: E13S basolat -> medium  $E13S*k1$ [nM/h] | k1 = 5.04 h^-1^ | [6, 20] |
| *v102* | Transport: E13S bile -> apical  $E13S*k1$ [nM/h] | k1 = 3.64 h^-1^ | [6, 20] |
| *v103* | Transport: E13S E13S hep -> apical  $E13S*k1$ [nM/h] | k1 = 3.64 h^-1^ | [6, 20] |
| *v104* | Transport: E13S hep -> basolat  $E13S*k1$ [nM/h] | k1 = 3.64 h^-1^ | [6, 20] |
| *v105* | Transport: E13S medium -> basolat  $E13S*k1$ [nM/h] | k1 = 3.64 h^-1^ | [6, 20] |
| *v106* | Transport: E13S hep -> bile; ABCG2  $\frac{E13S*kcat*ABCG2}{E13S+Km}$ [nM/h] | kcat = 150 h^-1^  km = 17000 nM | [40] |
| *v107* | Transport: E2 basolat -> hep  $E2*k1$ [nM/h] | k1 = 7.17 h^-1^ | [6, 20] |
| *v108* | Transport: E2 basolat -> medium  $E2*k1$ [nM/h] | k1 = 7.17 h^-1^ | [6, 20] |
| *v109* | Transport: E2 hep -> basolat  $E2*k1$ [nM/h] | k1 = 3.6 h^-1^ | [6, 20] |
| *v110* | Transport: E2 medium -> basolat  $E2*k1$ [nM/h] | k1 = 3.6 h^-1^ | [6, 20] |
| *v111* | Transport: E217βG apical -> bile  $E217\beta G*k1$ [nM/h] | k1 = 5.09 h^-1^ | [6, 20] |
| *v112* | Transport: E217βG apical -> hep  $E217\beta G*k1$ [nM/h] | k1 = 5.09 h^-1^ | [6, 20] |
| *v113* | Transport: E217βG basolat -> hep  $E217\beta G*k1$ [nM/h] | k1 = 5.09 h^-1^ | [6, 20] |
| *v114* | Transport: E217βG basolat -> medium  $E217\beta G*k1$ [nM/h] | k1 = 5.09 h^-1^ | [6, 20] |
| *v115* | Transport: E217βG bile -> apical  $E217\beta G*k1$ [nM/h] | k1 = 3.63 h^-1^ | [6, 20] |
| *v116* | Transport: E217βG hep -> apical  $E217\beta G*k1$ [nM/h] | k1 = 3.63 h^-1^ | [6, 20] |
| *v117* | Transport: E217βG hep -> basolat  $E217\beta G*k1$ [nM/h] | k1 = 3.63 h^-1^ | [6, 20] |
| *v118* | Transport: E217βG medium -> basolat  $E217\beta G*k1$ [nM/h] | k1 = 3.63 h^-1^ | [6, 20] |
| *v119* | Transport: E217βG E217BG hep -> bile; ABCG2  $\frac{E217\beta G*kcat*ABCG2}{E217\beta G+Km}$ [nM/h] | kcat = 372417 h^-1^  km = 44200 nM | [41] |
| *v120* | Transport: E217βG E217BG hep -> bile; MDR1  $\frac{E217\beta G*kcat*MDR1}{E217\beta G+Km}$ [nM/h] | kcat = 61981 h^-1^  km = 6200 nM | [42] |
| *v121* | Transport: E217βG E217BG hep -> bile; MRP2  $\frac{E217\beta G*kcat*MRP2}{E217\beta G+Km}$ [nM/h] | kcat = 1.9e+7 h^-1^  km = 98000 nM | [43] |
| *v122* | Transport: E217βG E217BG hep -> apical; MRP3  $\frac{E217\beta G*kcat*MRP3}{E217\beta G+Km}$ [nM/h] | kcat = 62976 h^-1^  km = 26000 nM | [44] |
| *v123* | Transport: E217βG E217BG hep -> apical; MRP4  $\frac{E217\beta G*kcat*MRP4}{E217\beta G+Km}$ [nM/h] | kcat = 305035 h^-1^  km = 30000 nM | [45] |
| *v124* | Transport: E23G basolat -> hep  $E23G*k1$ [nM/h] | k1 = 5.09 h^-1^ | [6, 20] |
| *v125* | Transport: E23G basolat -> medium  $E23G*k1$ [nM/h] | k1 = 5.09 h^-1^ | [6, 20] |
| *v126* | Transport: E23G hep -> basolat  $E23G*k1$ [nM/h] | k1 = 3.6 h^-1^ | [6, 20] |
| *v127* | Transport: E23G medium -> basolat  $E23G*k1$ [nM/h] | k1 = 3.6 h^-1^ | [6, 20] |
| *v128* | Transport: E23S basolat -> hep  $E23S*k1$ [nM/h] | k1 = 4.22 h^-1^ | [6, 20] |
| *v129* | Transport: E23S basolat -> medium  $E23S*k1$ [nM/h] | k1 = 4.22 h^-1^ | [6, 20] |
| *v130* | Transport: E23S hep -> basolat  $E23S*k1$ [nM/h] | k1 = 3.97 h^-1^ | [6, 20] |
| *v131* | Transport: E23S medium -> basolat  $E23S*k1$ [nM/h] | k1 = 3.97 h^-1^ | [6, 20] |
| *v132* | Transport: Diffusion limited access in medium: E2free = E2  $E2free*k1-E2*k2$ [nM/h] | k1 = 3600 h^-1^  k2 = 3600 h^-1^ | [12] |

## Balance Equations

| $\frac{\partial2MeE2(basolat)}{\partial t}$ | *v64 + v65 – v62 – v63* [nM/h] |
| --- | --- |
| $\frac{\partial2E217\beta G(basolat)}{\partial t}$ | *v117 + v118 – v113 – v114* [nM/h] |
| $\frac{\partial4OHE2(basolat)}{\partial t}$ | *v88 + v89 – v70 – v71* [nM/h] |
| $\frac{\partial2OHE2(basolat)}{\partial t}$ | *v72 + v73 – v62 – v63* [nM/h] |
| $\frac{\partial E2(basolat)}{\partial t}$ | *v109 + v110 – v107 – v108 – v31* [nM/h] |
| $\frac{\partial2E13G(basolat)}{\partial t}$ | *v96 + v97 – v94 – v95* [nM/h] |
| $\frac{\partial2E1(basolat)}{\partial t}$ | *v92 + v93 – v90 – v91* [nM/h] |
| $\frac{\partial E23S(basolat)}{\partial t}$ | *v130 + v131 – v128 – v129* [nM/h] |
| $\frac{\partial E13S(basolat)}{\partial t}$ | *v104 + v105 – v100 – v101* [nM/h] |
| $\frac{\partial E23G(basolat)}{\partial t}$ | *v126 + v127 – v124 – v125* [nM/h] |
| $\frac{\partial16\alpha OHE1(basolat)}{\partial t}$ | *v52 + v53 – v50 – v51* [nM/h] |
| $\frac{\partial2OHE1(basolat)}{\partial t}$ | *v68 + v69 – v66 – v67* [nM/h] |
| $\frac{16\alpha OHE2(basolat)}{\partial t}$ | *v56 + v57 – v54 – v55* [nM/h] |
| $\frac{\partial4OHE1(basolat)}{\partial t}$ | *v84 + v85 – v82 – v83* [nM/h] |
| $\frac{\partial4MeE2(basolat)}{\partial t}$ | *v80 + v81 – v78 – v79* [nM/h] |
| $\frac{\partial2MeE1(basolat)}{\partial t}$ | *v60 + v61 – v58 – v59* [nM/h] |
| $\frac{\partial4MeE1(basolat)}{\partial t}$ | *v76 + v77 – v74 – v75* [nM/h] |
| $\frac{\partial E217\beta G(apical)}{\partial t}$ | *v115 + v116 – v110 – v111* [nM/h] |
| $\frac{\partial E13S(apical)}{\partial t}$ | *v102 + v103 – v98 – v99* [nM/h] |
| $\frac{\partial E2(medium)}{\partial t}$ | *v108 + v132 – v110 – v46* [nM/h] |
| $\frac{\partial E1(medium)}{\partial t}$ | *v91 – v93 – v43* [nM/h] |
| $\frac{\partial E217\beta G(medium)}{\partial t}$ | *v142 + v122 + v123 – v118 – v47* [nM/h] |
| $\frac{\partial ER(medium)}{\partial t}$ | *– v31* [nM/h] |
| $\frac{\partial E2.ER(medium)}{\partial t}$ | *v31* [nM/h] |
| $\frac{\partial2MeE2(heps)}{\partial t}$ | *v62 + v4 – v63* [nM/h] |
| $\frac{\partial E23S(heps)}{\partial t}$ | *v128 + v29 – v130* [nM/h] |
| $\frac{\partial E13G(heps)}{\partial t}$ | *v94 + v12 + v13 – v96* [nM/h] |
| $\frac{\partial2OHE2(heps)}{\partial t}$ | *v70 + v18 + v19 – v72* [nM/h] |
| $\frac{\partial4OHE2(heps)}{\partial t}$ | *v86 + v20 + v21 – v88 – v6* [nM/h] |
| $\frac{\partial E2(heps)}{\partial t}$ | *v30 + v175 + v15 – v109 – v32 – v16 – v17 – v18 – v19 – v20 – v21 – v21 – v23 – v24 – v25 –v26 – v27 – v28 – v29* [nM/h] |
| $\frac{\partial E1(heps)}{\partial t}$ | *v90 + v22 + v23 – v92 – v7 – v8 – v9 – v10 – v11 – v12 – v13 – v15 – v14* [nM/h] |
| $\frac{\partial E13S(heps)}{\partial t}$ | *v99 + v100 + v14 – v103 – v104 – v105* [nM/h] |
| $\frac{\partial E217\beta G(heps)}{\partial t}$ | *v112 + v113 + v24 + v25 + v65 – v116 – v117 – v119 – v120 – v121 – v122 – v123* [nM/h] |
| $\frac{\partial E23G(heps)}{\partial t}$ | *v124 + v27 + v28 – v126* [nM/h] |
| $\frac{\partial16\alpha OHE1(heps)}{\partial t}$ | *v50 + v7 – v52* [nM/h] |
| $\frac{\partial2OHE1(heps)}{\partial t}$ | *v66 + v8 + v9 – v68 – v3* [nM/h] |
| $\frac{\partial16\alpha OHE2(heps)}{\partial t}$ | *v54 + v16 + v17 – v56* [nM/h] |
| $\frac{\partial4OHE1(heps)}{\partial t}$ | *v82 + v10 + v11 – v84 – v5* [nM/h] |
| $\frac{\partial4MeE2(heps)}{\partial t}$ | *v78 + v6 – v80* [nM/h] |
| $\frac{\partial2MeE1(heps)}{\partial t}$ | *v58 + v3 – v52* [nM/h] |
| $\frac{\partial4MeE1(heps)}{\partial t}$ | *v74 + v5 – v76* [nM/h] |
| $\frac{\partial E2.NSB(heps)}{\partial t}$ | *v40* [nM/h] |
| $\frac{\partial NSB(heps)}{\partial t}$ | *– v40* [nM/h] |

## Global Quantities

| **Parameter** | **Assignment** | **Ref** |
| --- | --- | --- |
| Biosynthesis Rate Constant | $Protein expression factor*6$ | * |
| Clearance: extra hepatic (CLeh) | $0$ |  |
| Protein Expression Factor | $0.1$ | [24, 46] |

* Biosynthesis rate constant was fitted to produce a steady-state estradiol concentration consistent with the experimental data

## Initial Conditions

| **Parameter** | **Assignment** | **Ref** |
| --- | --- | --- |
| ABCG2 (heps) | $83*Protein expression factor$ [nM] | [19, 47] |
| COMT (heps) | $596*Protein expression factor$ [nM] | [19, 47] |
| CYP1A2 (heps) | $13878*Protein expression factor$ [nM] | [19, 47] |
| CYP3A4 (heps) | $10219*Protein expression factor$ [nM] | [19, 47] |
| HSD17β1 (heps) | $541*Protein expression factor$ [nM] | [19, 47] |
| HSD17β2 (heps) | $1435*Protein expression factor$ [nM] | [19, 47] |
| MDR1 (heps) | $223*Protein expression factor$ [nM] | [19, 47] |
| MRP2 (heps) | $153*Protein expression factor$ [nM] | [19, 47] |
| MRP3 (heps) | $362*Protein expression factor$ [nM] | [19, 47] |
| MRP4 (heps) | $100*Protein expression factor$ [nM] | [19, 47] |
| SULT1E1 (heps) | $2932*Protein expression factor$ [nM] | [19, 47] |
| SULT2A1 (heps) | $64067*Protein expression factor$ [nM] | [19, 47] |
| UGT1A1 (heps) | $1980*Protein expression factor$ [nM] | [19, 47] |
| UGT1A3 (heps) | $2684*Protein expression factor$ [nM] | [19, 47] |
| UGT2B7 (heps) | $17959*Protein expression factor$ [nM] | [19, 47] |

# Quasi steady-state Petri net model of estrogen metabolism


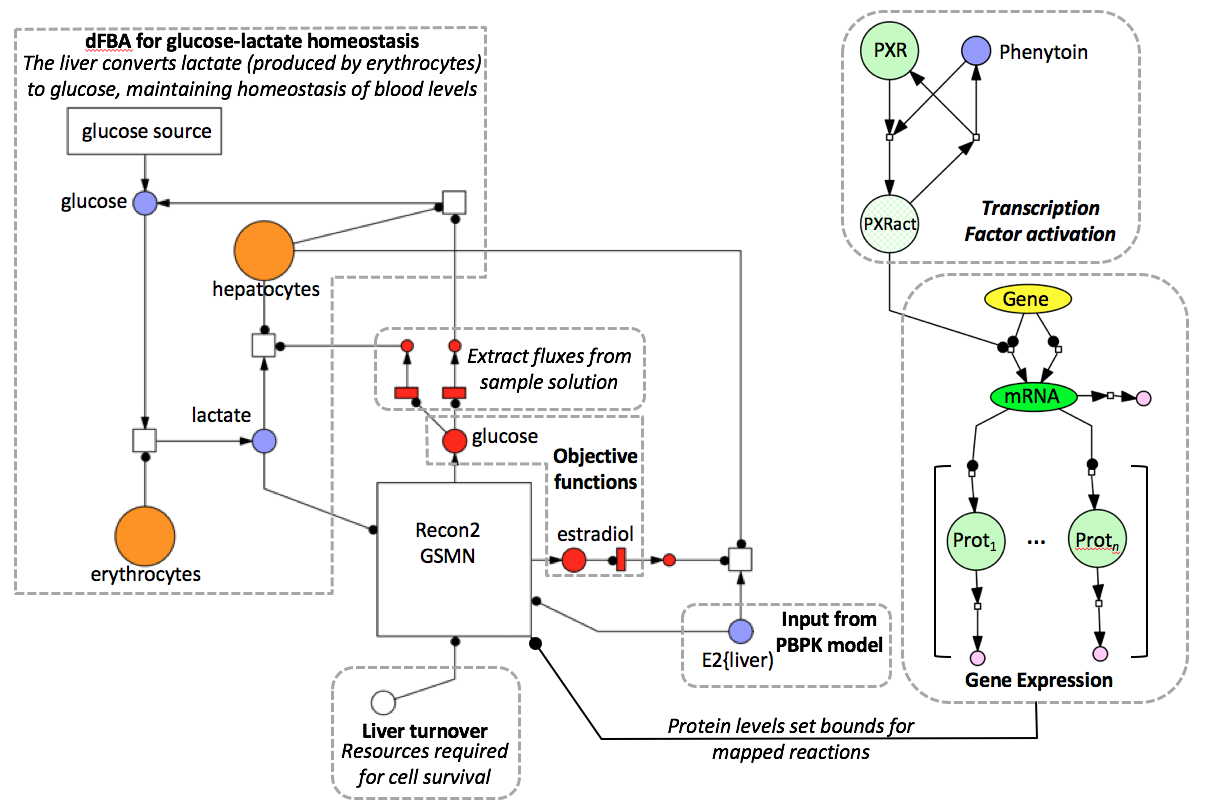


Figure S5: Quasi steady-state Petri net (QSSPN) model of estrogen metabolism. The model is constructed in Petri net formalism, where circles represent pre- and post- places (i.e. molecular species), and squares represent transitions (i.e. reactions). Places contain tokens that represent the number of a given molecular species. Places and transitions are connected in two ways: directed arcs (line with arrow head) denotes the movement of tokens from the pre-place to the post-place, representing metabolic conversions or transport; read arcs (line with filled circle) denotes the appearance of a token in the post-place that does not consume a token from the pre-place, representing catalysis or molecular interation for example. The model is divided into several functional units, indicated by the grey-hatched boxes. These represent signalling (transcription factor activation and gene expression), constraints (liver turnover and glucose-lactate dFBA), objective functions and connection with the PBPK model, which is also represented in Petric net formalism.

## Model Overview

In QSSPN, molecular interactions are assigned into one of two sets, dependent upon the timescale of operation: (1) fast reactions (i.e. millisecond timescale) are designated quasi-steady state fluxes, and are reconstructed using constraint-based modelling [48]. This set most commonly includes metabolic and transport reactions; (2) the dynamic transition set includes all reactions that occur over longer timescales (i.e. seconds to hours), and is reconstructed using an extended Petri net [49]; This set includes signalling pathways and gene regulation. As these two sets occur over different timescales, we assume a quasi steady-state due to timescale separation: effectivley, metabolic reactions occur in a series of steady-states, each one slightly differnt as informed by the gene and signal regulatory network reconstructed in the Petri net. For a full description of QSSPN, and its attendant software MuFINS, please see the accompanying papers [50, 51].

QSSPN models are constructed in Petri Net formalism using the SNOOPY graphical editor [52], which can be directly imported into the MuFINS software. The current model has a number of function units, indicated by the hatched grey boxes in figure S5. Gene regulation by phenytoin is reproduced through the interaction of phenyoin with the pregnane X-receptor (PXR), forming PXRact. This species then activates transcription and translation or PXR target genes, the expression level of which set bounds for mapped reactions in Recon2. Liver turnover is used to set a constraint on the biomass reaction, representing the minimum level of resources (e.g. amino acids, ATP, nucleotides etc.) required for the hepatocyte to be able to replenish itself. Objective functions set the reactions or metabolites that will be maximised during FBA. In this case, metabolites representing external glucose and estradiol degradation are used, with fluxes of interest extracted from the sample FBA solution to monitor the behaviour of the system. These fluxes are scaled by the number of hepatocytes present in a liver (or red blood cells) to reflect whole body physiology. The model is connected to the PBPK model through the species E2{liver}.

A major role of the liver is to buffer lactate levels in the blood, preventing acidosis. Red blood cells produce lactate through respiration, and this is secreted into the blood. The liver consumes lactate from the blood, converting it into glucose. The glucose objective within the QSSPN forms part of a dFBA module reproducting this behaviour. It adds an additional constraint whereby the GSMN must maintain glucose and lactate levels in the blood at 4mM and 1.5mM, respectively (Figure S5; [19]). A second important role of the liver is the detoxification of ammonia, a product of amino acide degradation. The liver converts ammonia to urea, which can then be excreted from the body in urine. The flux towards urea production is measured from the estradiol degradation objective function, and scaled by the number of hepatocytes to predict urea flux into the blood. Urea is removed through a transition representing excretion from the body, with the rate fitted to produce the known blood urea level, approximately 4mM (Figure S6b; [19]). Neither estrogen or phenytoin exposure impact on the predicted blood urea levels (data not shown).

Addition of phenytoin to the model results in activation of the nuclear receptor PXR, and increased expression of PXR target genes. Figure S6c shows that upon addition of 80μM phenytoin (time=0), an increase in target gene mRNA occurs, and is reaches a new steady state within approximately 4 hours. Protein levels also increase, approaching a new steady state by the end of the simulation period (48 hours). As protein levels pass the thresholds specificed within the QSSPN model, bounds for mapped reactions are increased. In the case of the PXR target gene CYP3A4, this increases the capacity of the metabolic network to convert estradiol to its catecholamine metabolites, leading to increases in the estradiol degradation flux (Figure S6c)

**A**

**B**

**C**

Figure S6: Simulation results for PBPK-GMSN model of estradiol in women. The response of the body to a 1mg oral dose of estradiol was simulated over 48 hours and the levels of (A) estradiol and (B) glucose, lactate and urea predicted in the blood. (C) The simulation was re-run with the addition of 80μM phenytoin. mRNA and protein levels of the PXR target gene CYP3A4 were predicted, as well as the flux towards estradiol degradation

# Anthropometric Data

## Overview

To generate a virtual patient population, data was extracted from the 2013-14 U.S. National Health and Nutrition Examination Survey (NHANES [53]). Data for 1495 females were extracted using the age range 18-45 inclusive, to represent a post-pubescent but pre-menopausal population. The first ten of these were selected to act as the virtual patient population. The anthropometric data for age, weight and height did not differ significantly between this sub-population and the total dataset. We do note, however, that the mean weight is significantly higher than the commonly used 60kg for a female, being 75.7kg. In addition, the distribution is significantly non-normal. These represent a modern U.S. population, and reflect the increase in obese individuals within this population during the last few decades.

##
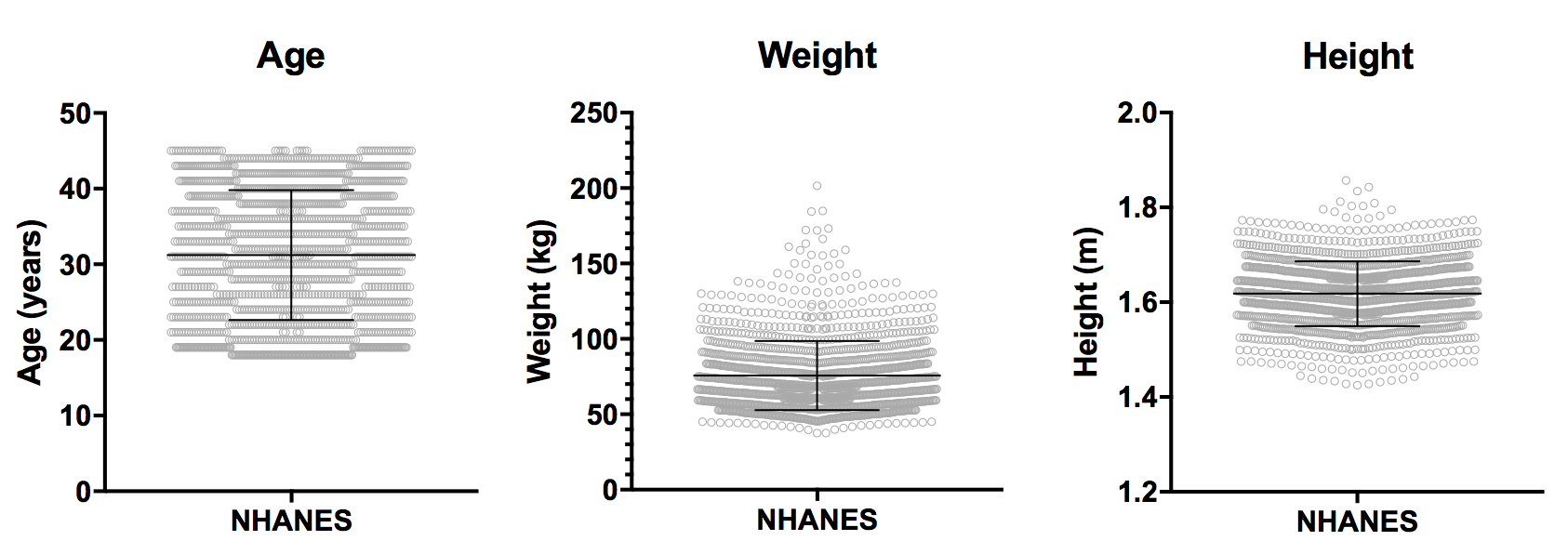


Figure S7: Summary anthropometric statistics for NHANES 2013-14. Anthropometric data for age, weight and height for females 18-45 were extracted from the 2013-14 NHANES. Individual values are presented, along with the population mean (± s.d.)

**References**

1. Hoops S, Sahle S, Gauges R, Lee C, Pahle J, Simus N, Singhal M, Xu L, Mendes P, Kummer U: **COPASI — a COmplex PAthway SImulator**. *Bioinformatics* 2006, **22**:3067-3074.

2. Plowchalk DR, Teeguarden J: **Development of a physiologically based pharmacokinetic model for estradiol in rats and humans: A biologically motivated quantitative framework for evaluating responses to estradiol and other endocrine-active compounds**. *Toxicol Sci* 2002, **69**(1):60-78.

3. Bosgra S, van Eijkeren J, Bos P, Zeilmaker M, Slob W: **An improved model to predict physiologically based model parameters and their inter-individual variability from anthropometry**. *Crit Rev Toxicol* 2012, **42**(9):751-767.

4. Tulchins.D, Yeager E, Hobel CJ, Marshall JR: **Plasma estrone, estradiol, estriol, progesterone, and 17-hydroxyprogesterone in human pregnancy .1. Normal pregnancy**. *Am J Obstet Gynecol* 1972, **112**(8):1095-&.

5. Stricker R, Eberhart R, Chevailler M-C, Quinn FA, Bischof P, Stricker R: **Establishment of detailed reference values for luteinizing hormone, follicle stimulating hormone, estradiol, and progesterone during different phases of the menstrual cycle on the Abbott ARCHITECT (R) analyzer**. *Clin Chem Lab Med* 2006, **44**(7):883-887.

6. Wishart DS, Jewison T, Guo AC, Wilson M, Knox C, Liu Y, Djoumbou Y, Mandal R, Aziat F, Dong E *et al*: **HMDB 3.0-The Human Metabolome Database in 2013**. *Nucleic Acids Res* 2013, **41**(D1):D801-D807.

7. Peters SA: **Evaluation of a generic physiologically based pharmacokinetic model for lineshape analysis**. *Clinical Pharmacokinetics* 2008, **47**(4):261-275.

8. Murphy JE, Janszen DB, Gargas ML: **An in-vitro method for determination of tissue partition-coefficients of nonvolatile chemicals such as 2,3,7,8-tetrachlorodibenzo-p-dioxin and estradiol**. *J Appl Toxicol* 1995, **15**(2):147-152.

9. Sodergard R, Backstrom T, Shanbhag V, Carstensen H: **Calculation of free and bound fractions of testosterone and estradiol-17beta to human plasma proteins at body temperature**. *Journal of Steroid Biochemistry and Molecular Biology* 1982, **16**(6):801-810.

10. Benard F, Ahmed N, Beauregard JM, Rousseau J, Aliaga A, Dubuc C, Croteau E, van Lier JE: **F-18 fluorinated estradiol derivatives for oestrogen receptor imaging: impact of substituents, formulation and specific activity on the biodistribution in breast tumour-bearing mice**. *Eur J Nucl Med Mol Imag* 2008, **35**(8):1473-1479.

11. Deurenberg P, Weststrate JA, Seidell JC: **Body-mass index as a measure of body fatness - Age-specific and sex-specific prediction formulas**. *Br J Nutr* 1991, **65**(2):105-114.

12. Kolodkin AN, Bruggeman FJ, Plant N, Mone MJ, Bakker BM, Campbell MJ, van Leeuwen JPTM, Carlberg C, Snoep JL, Westerhoff HV: **Design principles of nuclear receptor signaling: how complex networking improves signal transduction**. *Mol Sys Biol* 2010, **6**:446.

13. Khan N, Sharma KK, Andersson S, Auchus RJ: **Human 17 beta-hydroxysteroid dehydrogenases types 1, 2, and 3 catalyze bi-directional equilibrium reactions, rather than unidirectional metabolism, in HEK-293 cells**. *Arch Biochem Biophys* 2004, **429**(1):50-59.

14. Mizrachi D, Auchus RJ: **Androgens, estrogens, and hydroxysteroid dehydrogenases**. *Mol Cell Endocrinol* 2009, **301**(1-2):37-42.

15. Yamazaki H, Shaw PM, Guengerich FP, Shimada T: **Roles of cytochromes P450 1A2 and 3A4 in the oxidation of estradiol and estrone in human liver microsomes**. *Chem Res Toxicol* 1998, **11**(6):659-665.

16. Tsuchiya Y, Nakajima M, Yokoi T: **Cytochrome P450-mediated metabolism of estrogens and its regulation in human**. *Cancer Letters* 2005, **227**(2):115-124.

17. McNamara KM, Nakamura Y, Miki Y, Sasano H: **Phase two steroid metabolism and its roles in breast and prostate cancer patients**. *Frontiers in endocrinology* 2013, **4**:116-116.

18. Dawling S, Roodi N, Mernaugh RL, Wang XH, Parl FF: **Catechol-O-methyltransferase (COMT)-mediated metabolism of catechol estrogens: Comparison of wild-type and variant COMT isoforms**. *Cancer Res* 2001, **61**(18):6716-6722.

19. Kolker E, Higdon R, Haynes W, Welch D, Broomall W, Lancet D, Stanberry L, Kolker N: **MOPED: Model Organism Protein Expression Database**. *Nucleic Acids Res* 2012, **40**(D1):D1093-D1099.

20. Vandewaterbeemd H, Vanbakel P, Jansen A: **Transport in quantitative structure-activity-relationships:6. Relationship between transport rate constants and partition-coefficients**. *Journal of Pharmaceutical Sciences* 1981, **70**(9):1081-1082.

21. Tetko IV, Tanchuk VY: **Application of associative neural networks for prediction of lipophilicity in ALOGPS 2.1 program**. *J Chem Inf Comput Sci* 2002, **42**(5):1136-1145.

22. Leach AR, Shoichet BK, Peishoff CE: **Prediction of protein-ligand interactions. Docking and scoring: Successes and gaps**. *Journal of Medicinal Chemistry* 2006, **49**(20):5851-5855.

23. Guo T, Gu J, Soldin OP, Singh RJ, Soldin SJ: **Rapid measurement of estrogens and their metabolites in human serum by liquid chromatography-tandem mass spectrometry without derivatization**. *Clin Biochem* 2008, **41**(9):736-741.

24. Padgham C, Paine A: **Altered expression of cytochrome P450 mRNA's, and potentially of other transcripts encoding key hepatic functions, are triggered during the isolation of rat hepatocytes**. *Biochem J* 1993, **289**:621-624.

25. Plant N: **Strategies for using *in vitro* screens in drug metabolism**. *Drug Discov Today* 2004, **9**(7):328-336.

26. Dawling S, Hachey DL, Roodi N, Parl FF: **In vitro model of mammary estrogen metabolism: Structural and kinetic differences between catechol estrogens 2-and 4-hydroxyestradiol**. *Chem Res Toxicol* 2004, **17**(9):1258-1264.

27. Zhu BT, Wang P, Nagai M, Wen Y, Bai H-W: **Inhibition of human catechol-O-methyltransferase (COMT)-mediated O-methylation of catechol estrogens by major polyphenolic components present in coffee**. *Journal of Steroid Biochemistry and Molecular Biology* 2009, **113**(1-2):65-74.

28. Shou MG, Korzekwa KR, Brooks EN, Krausz KW, Gonzalez FJ, Gelboin HV: **Role of human hepatic cytochrome P450 1A2 and 3A4 in the metabolic activation of estrone**. *Carcinogenesis* 1997, **18**(1):207-214.

29. Jefcoate CR, Liehr JG, Santen RJ, Sutter TR, Yager JD, Yue W, Santner SJ, Tekmal R, Demers L, Pauley R *et al*: **Chapter 5: Tissue-specific synthesis and oxidative metabolism of estrogens**. *Journal of the National Cancer Institute Monographs* 2000(27):95-112.

30. Lepine J, Bernard O, Plante M, Tetu B, Pelletier G, Labrie F, Belanger A, Guillemette C: **Specificity and regioselectivity of the conjugation of estradiol, estrone, and their catecholestrogen and methoxyestrogen metabolites by human uridine diphospho-glucuronosyltransferases expressed in endometrium**. *J Clin Endocr Metab* 2004, **89**(10):5222-5232.

31. Schrag ML, Cui DH, Rushmore TH, Shou MG, Ma B, Rodrigues AD: **Sulfotransferase 1E1 is a low K-M isoform mediating the 3-O-sulfation of ethinyl estradiol**. *Drug Metab Dispos* 2004, **32**(11):1299-1303.

32. Gangloff A, Garneau A, Huang YW, Yang F, Lin SX: **Human oestrogenic 17 beta-hydroxysteroid dehydrogenase specificity: enzyme regulation through an NADPH-dependent substrate inhibition towards the highly specific oestrone reduction**. *Biochemical Journal* 2001, **356**:269-275.

33. Usmani KA, Cho TM, Rose RL, Hodgson E: **Inhibition of the human liver microsomal and human cytochrome P450 1A2 and 3A4 metabolism of estradiol by deployment-related and other chemicals**. *Drug Metab Dispos* 2006, **34**(9):1606-1614.

34. Williams JA, Ring BJ, Cantrell VE, Jones DR, Eckstein J, Ruterbories K, Hamman MA, Hall SD, Wrighton SA: **Comparative metabolic capabilities of CYP3A4, CYP3A5, and CYP3A7**. *Drug Metab Dispos* 2002, **30**(8):883-891.

35. Gale EAM: **Lessons from the glitazones: a story of drug development**. *Lancet* 2001, **357**(9271):1870-1875.

36. Blomquist CH: **Kinetic analysis of enzymic activities: Prediction of multiple forms of 17 beta-hydroxysteroid dehydrogenase**. *Journal of Steroid Biochemistry and Molecular Biology* 1995, **55**(5-6):515-524.

37. Lu ML, Huang YW, Lin SX: **Purification, reconstitution, and steady-state kinetics of the trans-membrane 17 beta-hydroxysteroid dehydrogenase 2**. *J Biol Chem* 2002, **277**(25):22123-22130.

38. Wang LQ, James MO: **Sulfotransferase 2A1 forms estradiol-17-sulfate and celecoxib switches the dominant product from estradiol-3-sulfate to estradiol-17-sulfate**. *Journal of Steroid Biochemistry and Molecular Biology* 2005, **96**(5):367-374.

39. Riches Z, Stanley EL, Bloomer JC, Coughtrie MWH: **Quantitative Evaluation of the Expression and Activity of Five Major Sulfotransferases (SULTs) in Human Tissues: The SULT "Pie"**. *Drug Metab Dispos* 2009, **37**(11):2255-2261.

40. Suzuki M, Suzuki H, Sugimoto Y, Sugiyama Y: **ABCG2 transports sulfated conjugates of steroids and xenobiotics**. *J Biol Chem* 2003, **278**(25):22644-22649.

41. Chen ZS, Robey RW, Belinsky MG, Shchaveleva I, Ren XQ, Sugimoto Y, Ross DD, Bates SE, Kruh GD: **Transport of methotrexate, methotrexate polyglutamates, and 17 beta-estradiol 17-(beta-D-glucuronide) by ABCG2: Effects of acquired mutations at R482 on methotrexate transport**. *Cancer Res* 2003, **63**(14):4048-4054.

42. Huang L, Hoffman T, Vore M: **Adenosine triphosphate-dependent transport of estradiol-17beta(beta-D-glucuronide) in membrane vesicles by MDR1 expressed in insect cells**. *Hepatology* 1998, **28**(5):1371-1377.

43. Pedersen JM, Matsson P, Bergstrom CAS, Norinder U, Hoogstraate J, Artursson P: **Prediction and identification of drug interactions with the human ATP-binding cassette transporter multidrug-resistance associated protein 2 (MRP2; ABCC2)**. *Journal of Medicinal Chemistry* 2008, **51**(11):3275-3287.

44. Howe K, Sanat F, Thumser AE, Coleman T, Plant N: **The statin class of HMG-CoA reductase inhibitors demonstrate differential activation of the nuclear receptors PXR, CAR and FXR, as well as their downstream target genes**. *Xenobiotica* 2011, **41**(7):519-529.

45. Chen ZS, Lee K, Kruh GD: **Transport of cyclic nucleotides and estradiol 17-beta-D-glueuronide by multidrug resistance protein 4 - Resistance to 6-mercaptopurine and 6-thioguanine**. *J Biol Chem* 2001, **276**(36):33747-33754.

46. Padgham CRW, Paine AJ, Phillips IR, Shephard EA: **Maintenance of total cytochrome P450 content in rat hepatocyte culture and the abundance of CYP1A2 and CYP2B1/2 mRNA's**. *Biochem J* 1992, **285**:929-932.

47. Kim M-S, Pinto SM, Getnet D, Nirujogi RS, Manda SS, Chaerkady R, Madugundu AK, Kelkar DS, Isserlin R, Jain S *et al*: **A draft map of the human proteome**. *Nature* 2014, **509**(7502):575-+.

48. Covert MW, Famili I, Palsson BO: **Identifying constraints that govern cell behavior: A key to converting conceptual to computational models in biology?** *Biotechnology and Bioengineering* 2003, **84**(7):763-772.

49. Breitling R, Gilbert D, Heiner M, Orton R: **A structured approach for the engineering of biochemical network models, illustrated for signalling pathways**. *Brief Bionfor* 2008, **9**(5):404-421.

50. Fisher CP, Plant NJ, Moore JB, Kierzek AM: **QSSPN: Dynamic Simulation of Molecular Interaction Networks Describing Gene Regulation, Signalling and Whole-Cell Metabolism in Human Cells**. *Bioinformatics* 2013, **29**(24):3181-3190.

51. Wu H, von Kamp A, Leoncikas V, Mori W, Sahin N, Gevorgyan A, Linley C, Grabowski M, Mannan AA, Stoy N *et al*: **MUFINS: Multi-Formalism Interaction Network Simulator**. *npg Systems Biology and Applications* 2016, ***2***:16032.

52. Rohr C, Marwan W, Heiner M: **Snoopy-a unifying Petri net framework to investigate biomolecular networks**. *Bioinformatics* 2010, **26**(7):974-975.

53. **National Health and Nutrition Examination Survey Data** [<http://www.cdc.gov/nchs/nhanes.htm>]
